# Stability and Properties of Ultraviolet Filter Avobenzone under Its Diketo/Enol Tautomerization Induced by Molecular Encapsulation with β-Cyclodextrin

**DOI:** 10.1021/acs.langmuir.4c04108

**Published:** 2025-01-07

**Authors:** Chihiro Kuroda, Tomohiro Tsuchida, Chihiro Tsunoda, Megumi Minamide, Ryosuke Hiroshige, Satoru Goto

**Affiliations:** Faculty of Pharmaceutical Sciences, Tokyo University of Science, 2641 Yamazaki, Noda, Chiba 278-8510, Japan

## Abstract

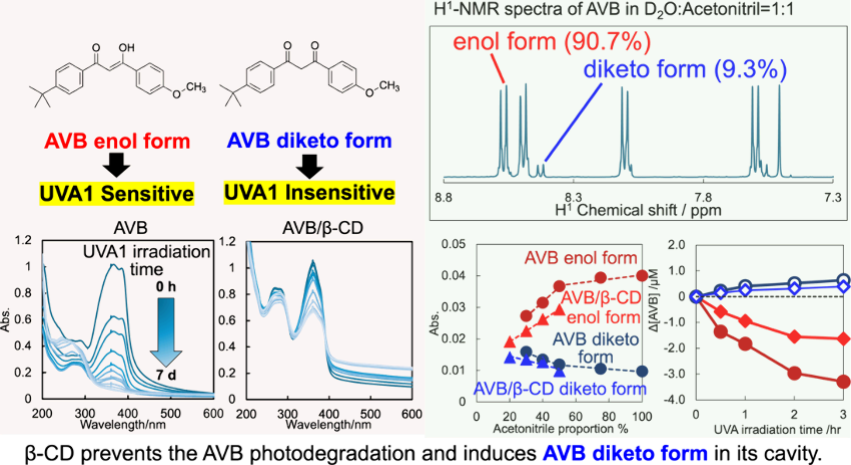

Inclusion complexation of the sunscreen ingredient avobenzone (AVB) with β-cyclodextrin (β-CD) was investigated to improve its aqueous solubility and photostability; another ultraviolet (UV) filter, oxybenzone (OXB), and the phytochemical antioxidant curcumin (CUR) served as a comparison. In this study, the 1-octanol/water partition coefficients, acid dissociation constants, phase-solubility diagrams with β-CD, and ultraviolet–visible (UV–vis) spectral changes induced by UVA1 (365 nm) irradiation were evaluated. β-CD at concentrations 50–100 times that of AVB most effectively protected the photostability of AVB. Additionally, an UVA1-insensitive species with a diketo tautomer, which has an UVC-absorbing band and the potential to cause photodegradation, was stored in the inclusion complex. Acetonitrile–water mixtures at various volume ratios were screened to mimic the internal cavity of β-CD for the AVB tautomeric species using nuclear magnetic resonance (NMR) spectral integrals for the components. The results indicated that β-CD provides a hydrophobic environment similar to that of a 40–50% acetonitrile aqueous solution and enhances the photostability of AVB. However, excess β-CD induced a hyperchromic effect on the diketo tautomer. Aggregation of the AVB/β-CD inclusion complexes at β-CD concentrations of ≥2 mM enhances UVC band absorption. To avoid excess β-CD, a molar ratio of 50–100 of β-CD to AVB is recommended as the optimal composition. This study newly exhibited that the cavity of β-CD mitigates the reactivity of UVA1 toward AVB by inducing the diketo tautomer form of AVB within the cavity.

## 1 Introduction

The United States Food and Drug Administration (U.S. FDA) began to use the term “generally recognized as safe and effective” (GRASE) for over-the-counter (OTC) drugs and ingredients in therapeutic products.^1−3^ The GRASE ingredients numbered in the hundreds and included many familiar products, such as sunscreen, pain relievers, and medicated lotions. On the basis of scientific evidence and clinical studies, the U.S. FDA reverted to the 2019 proposal that an OTC drug product is safe and effective for its intended use.^4^

**Chart 1:**
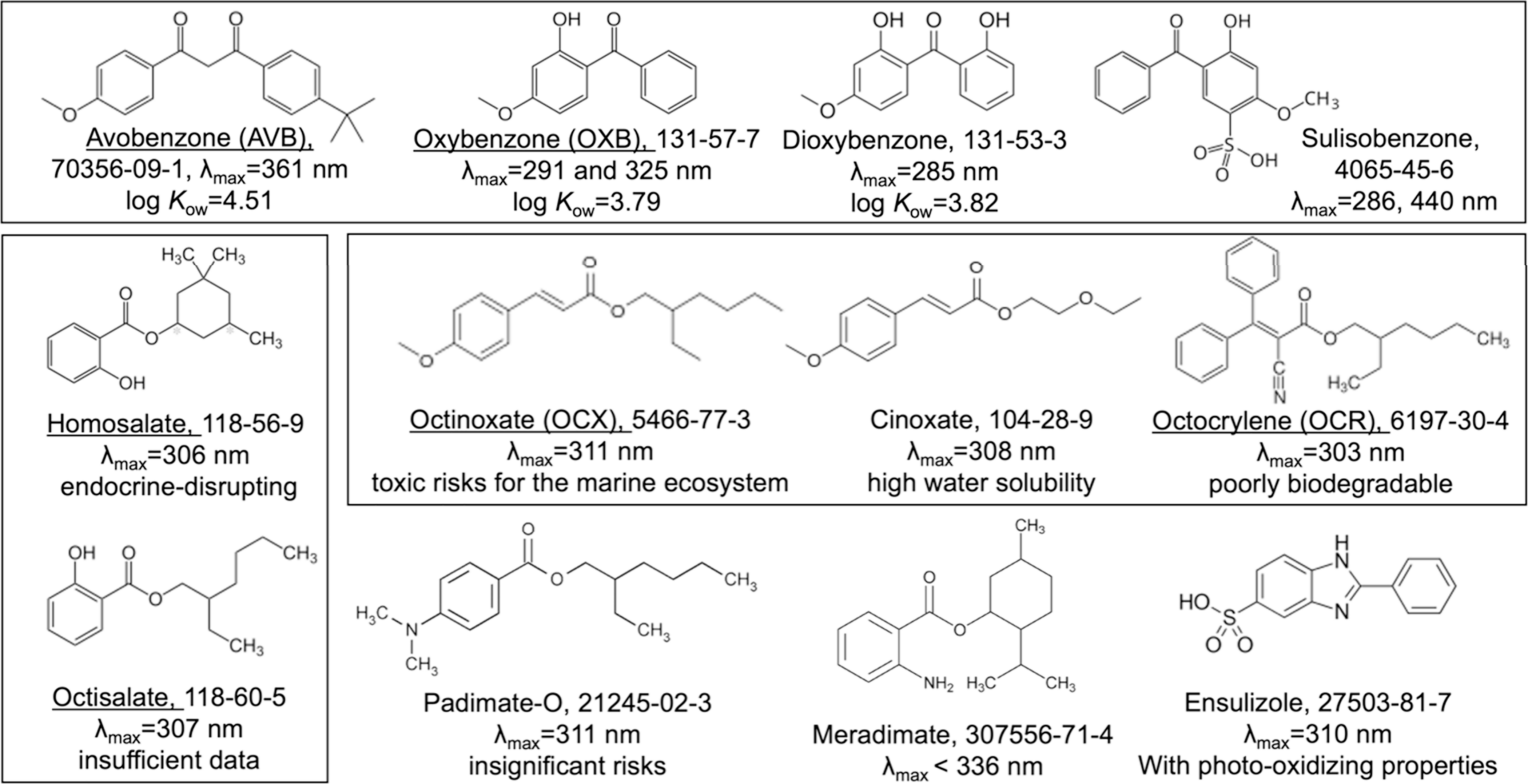
Chemical Structures and Properties of the Dominant Chemical UV Filter Ingredients^5^

The U.S. FDA proposed its latest regulatory update for the products to oversee sunscreen safety in 2019.^4^ On the basis of the available information, the agency reviewed 16 ingredients and reported that only two physical/inorganic/mineral products, ZnO and TiO_2_, were GRASE. In contrast, the following chemical/organic/synthetic ultraviolet (UV) filters are non-GRASE ingredients because of insufficient data (see Chart 1): the benzophenone derivatives, e.g., avobenzone [AVB, *p*-*tert*-butyl-*p*-methoxydibenzoylmethane (BMDM)], oxybenzone [OXB, benzophenone-3 (BP-3), 2-hydroxy-4-methoxyphenylbenzophenone (HMBP)], dioxybenzone (benzophenone-8), and sulisobenzone (benzophenone-4), the salicylate esters, e.g., homosalate and octisalate, the cinnamoyl esters, e.g., octocrylene (OCR), octinoxate (OCX), and cinoxate, and others, e.g., padimate O, meradimate, and ensulizole. *para*-Aminobenzoic acid and trolamine salicylate are no longer used in sunscreen marketed in the U.S.^5^

AVB, OXB, and homosalate are considered endocrine-disruptors.^6^ The U.S. FDA has reported that they and other ingredients, such as octisalate, OCX, and OCR, are systemically absorbed into the body and can be detected in the skin, blood plasma, breast milk, and urine samples weeks after use.^7^ The European Commission has ruled on the safety of homosalate and OCR, and, have proposed to limit allowable usage concentrations.^8,9^ This ruling does not imply that the other aforementioned UV filters are excluded.

These rules may not be well received by those with darker colored skin. The demand for organic UV filters is a testament to public concerns and preferences. Customers avoid sunscreen products containing inorganic UV-scattering agents, claiming that whiteness affects their makeup and appearance. The demand for organic UV filters is a significant factor in the sunscreen market. The maximum allowed concentration of OCX is 7.5% in the U.S. and Canada, whereas OCX is regulated at 10% in the European Union (EU), People’s Republic of China (PRC), and Australia.^10−13^ In Japan, an OCX concentration as high as 20% is accepted. In contrast, OCX-based products are banned in Hawaii because of their toxicity to marine ecosystems.^14^

We aimed to explore a strategy that enhances the aqueous solubility of hydrophobic ingredients or topical antidrugs to avoid transdermal adsorption into the blood.^15−25^ In addition, we aimed to clarify the molecular mechanism by which UVA (320–400 nm, frequently pouring regardless of climate) absorption and photodegradation of the benzophenone-type UV filter AVB are protected and sustained by the aqueous solubilizing reagent, cyclodextrins (CDs), which encapsulate ligands into their hydrophobic internal cavity and restrict tautomerization and degradation.^26−29^

AVB and OXB are favorable examples of UV filters because their spectroscopic traceability allows for physicochemical experiments.^29^ AVB, the most prominent UVA filter, is known for its ability to absorb a wide range of UVA rays, particularly in the UVA1 band (340–400 nm). Sunscreen formulations containing AVB provide broad-spectrum protection from UV exposure and are key ingredients in popular products. AVB is approved for use with maximum concentrations ranging from 3% in the U.S. and Canada to 5% in the EU and Australia.^10−14^

Given Lipinski’s rule of five,^30^ AVB with a partition coefficient of log *K*_OW_ = 4.51 and molecular weight of 310.4 g/mol is expected, with a high skin permeability from 1.8 to 4.3 ng/mL.^31^ The keto-enol form with intramolecular hydrogen bonding (the chelated keto-enol form) is coplanar, photostable, and absorbs in the UVA1 band. In contrast, the diketo tautomer absorbs light in the UVC range (200–280 nm) and is prone to degradation.^32^ The efficacy of the keto-enol form as a barrier for UVA exposure and protection against skin cancer is reduced because tautomerism between the keto-enol and diketo forms leads to instability and harm.^33,34^ Notably, the photodegradation products of avobenzone, which are highly reactive radical species, potentially induce inflammation in skin tissue and have harmful effects on human health.^35^

Modifications to the scaffold are required to overcome these drawbacks of AVB; the modifications include swapping AVB aromatic groups, prohibiting aggregation and avoiding diketo formation with alternatives.^36^ Other methods for retaining the AVB structure include quenching the triplet excited state using chemical additives,^37,38^ scavenging radicals using antioxidants,^39^ and encapsulation in micelles/cyclodextrin/metal complexes.^40^ Electron density at the diketo group can be an efficient target to regulate the photodegradation process of AVB owing to α-cleavage of the diketo form with a triplet excited state via a Norrish type I reaction.^34,35,37^

## 2 Materials and Methods

### 2.1 Materials

AVB (CAS Registry Number 70356-09-1) was supplied by Fujifilm Wako Pure Chemical Industries (Osaka, Japan). OXB (131-57-7), curcumin (CUR, 458-37-7), β-CD (7585-39-9), hydroxypropyl-β-cyclodextrin (HP-β-CD, 128446-35-5), HPLC-grade 1-octanol, and deuterated solvents (D_2_O, methanol-*d*_4_, acetonitrile-*d*_3_, and other solvents) were obtained from Tokyo Chemical Industry (Tokyo, Japan). All of the other materials and solvents were of analytical grade.

The aqueous-phase solvents were prepared by mixing 25 mM KH_2_PO_4_ and 25 mM Na_2_HPO_4_ solutions in 25 mM P_i_ buffer. The pH was adjusted before fixing the prescribed concentrations of the acid components of the buffer. The JP1 and JP2 buffer solutions were 10 mM sodium citrate/HCl buffer (pH 1.2) and P_i_ buffer (pH 6.8), which were prepared in compliance with the description for solution media for the dissolution test in Japanese Pharmacopeia (JP)-implemented international harmonization. The modified Britton–Robinson universal pH buffer comprised 28.6 mM KH_2_PO_4_, 28.6 mM HBO_2_, and 28.6 mM NaCl, titrated with 1 M NaOH (here, modified means elimination of barbital to cancel its interaction with organic solutes). For the partition equilibrium examination, the aqueous saturated 1-octanol phase comprised 1-octanol, which was flooded with a modified Britton–Robinson buffer adjusted to the appropriate pH in advance.

### 2.2 Reversed-Phase High-Performance Liquid Chromatography (HPLC) Measurements

The sample solution was filtered with a membrane filter (Minisart RC 4, 0.22 μm pore size, Sartorius, Göttingen, Germany). Fractionation of the sample in the filtrate was performed by HPLC (SPD-20A, Shimadzu Co., Kyoto, Japan) with a mobile phase of 25 mM citric acid buffer (pH 3.0)/methanol (3:7) or 25 mM P_i_ buffer (pH 2.5)/acetonitrile-*d*_3_ (1:1) at a flow rate of 1 mL/min, using a reversed-phase column (Capcell Pak C18, Shiseido, 5 μm, 250 mm × ⌀ 4.6 mm) at a temperature of 313 K. The concentrations of AVB, OXB, and CUR were determined by monitoring with a photodiode array (PDA) detector at wavelengths between 200 and 600 nm. A single-beam detector was used at wavelengths of 360 and 270 nm for AVB and 430 and 230 nm for CUR.

### 2.3 Flask-Shaking Method for the 1-Octanol/Water Partition Experiment

The hydrophobic drug dissolved in the aqueous saturated 1-octanol phase was mixed with an isochoric solution of the modified Britton–Robinson buffer (pH 5–11.5), saturated with 1-octanol. Thereafter, the samples in the screw-capped vials were machine-shaken at 298 K in a thermostatic chamber for 60 min and subsequently kept stationary for 60 min; the procedure corresponds to the conventional “flask-shaking” method.^41−43^ After settling, the pH in the aqueous phase was confirmed using pH indicator strips (MColoHast pH 0–6.0, MQuant pH 7.5–14, and MQuant pH 0–14, Merck, Germany) to be that of the adjusted pH value. Aliquots (10 μL) from the 1-octanol and the aqueous layers were injected into the HPLC instrument. The 1-octanol/water partition coefficients, log *P* (equivalent to log *K*_OW_), and acid dissociation constants, p*K*_a_, were optimized using curve fitting, approximated by eq 1
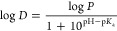
1which is Avdeef’s diagram^44^ derived from the Henderson–Hasselbalch expression for the equilibrium between the protonated and deprotonated species. The curve-fitting procedures used the solver module of Microsoft Excel 2016 with the implemented GRG nonlinear option. Approximate curves corresponding to these optimized parameters were drawn, and the p*K*_a_ and log *P* values for AVB, OXB, and CUR were obtained.

### 2.4 Phase-Solubility Isotherm Diagram of Drug to CDs

An excess amount of drug powder, coordinated by groping trials, was added to screw-capped vials containing the P_i_ buffer (5 mL, pH 6.8) in the absence or presence of β-CD or HP-β-CD. The solutions were mechanically shaken at 298 K in a thermostatic chamber, and the supernatant was sampled and filtered after passage for appropriate periods. The concentration of the drug in the supernatant was determined using HPLC.

The phase-solubility diagram consists of the equilibrium concentration of the guest drug as the ordinate and the total concentration of the host CD as the abscissa.^45^ If a straight line and parabolic curve can be approximated on the phase-solubility diagram, then, the curves are classified as A_L_- and A_P_-types in the Higuchi and Connors catalogs, respectively.^45,46^ For the linear correlation corresponding to the A_L_-type, regression analysis of the experimental data set using eq 2 provides the stability constant *K*_1:1_ for equimolar drug/CD complexes
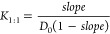
2where *D*_0_ is the solubility of the drug in the absence of CD, which is comparable to the ordinate intercept, and the slope is the gradient of the ordinate, referred to as the abscissa.

The parabolic curve corresponding to the A_P_-type indicated that the complexes were associated with a stoichiometry of equimolar guest/host and that of single guest/double hosts (or further convoluted proportions).^45,46^ Multiple regression analysis of the measured data set to the total concentration of the host ([CD]) and its squares ([CD]^2^) using eq 3 can be used to obtain the stability constants for a combination of the equimolar complex *K*_1:1_ and double-host associating complex *K*_1:2_.^45,46^
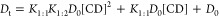
3This is the sum of the dissociated (free) and associated (complexed) drug concentrations. Although the Benesi–Hildebrand equation with the square of the host concentration has been used for parabolic correlations, its validity remains unclear.

Occasionally, a phase-solubility diagram exhibits a hyperbolic curve, referred to as a saturation curve. This curve is classified as the A_N_-type on the Higuchi and Connors catalog.^45−48^ The curve is described by eq 4′ according to the Langmuir adsorption isotherm model.^47−51^ To process with the linear regression analysis of ordinate ([CD]/γ) referred to abscissa ([CD]), the Hanes–Woolf expression in eq 4′ may be used
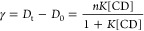
4
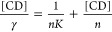
4′where *n* indicates the asymptotic concentration of the drug associated with CD (saturation, maximal γ) and *K* is equivalent to the drug/CD complex stability constant. If the concentration of CD is comparable to reciprocal *K*, then the obtained γ is accorded to the half amount of *n*. Notably, the saturated curve illustrates that the solubility of the guest–host complex is less potent and that the association is more complicated.^46^ The stoichiometry of a complex of drugs with CD is difficult to determine; the prevailing opinion is that more than one guest molecule incorporates a host molecule and that hydration or ionization of the guest regulates the solubility of the complex.^46^

Szejtili^46^ stated that, in rare cases, the ascending region at lower host concentrations is followed by a plateau region, similar to that of the A_N_-type saturation curve. Simultaneously, it terminates at any host concentration threshold. It is named the B_S_-type in the Higuchi and Connors catalog,^45−48^ and for concentrations higher than the threshold, the guest concentration decreases along a hyperbola, probably according to the solubility product *K*_SP_.^49^ To analyze this type of pattern, a hypothesis that is yet to be discussed is required.^52^ For the concentration of the drug in the plateau region, a soluble complex of the drug and CD is enlarged by adhesion of the excess CD, similar to a snowball. At the threshold concentration of CD, the scale of the aggregated dispersoids transcends an upper limit, and the aggregation induces subsequent sedimentation.^52^ Thus, the solubility product may be considered a property of poorly soluble aggregated dispersoids containing a tolerable amount of the drug and an excess amount of CD.

### 2.5 UV–Vis Spectroscopy for CD Inclusion and UVA1 Photodegradation

UVA1 (365 nm) was dissolved in the 25 mM P_i_ buffer (pH 6.8) in the absence or presence of β-CD and was uniformly irradiated onto samples using an UV lamp (GL15, Toshiba Co., Tokyo, Japan, 15 W) at an optical path length of 15 cm. The samples were obtained after passage for appropriate periods and measured using an UV–vis spectrophotometer in the maximum range between 200 and 700 nm (arranged on demand) from long wavelengths toward short wavelengths to avoid unnecessary irradiation of the UV region. To obtain HPLC chromatograms, aliquots of the UV-irradiated solution were obtained by pipetting at 0, 1/5, 1/2, 1, 2, 3, 4, 24, 48, 72, 96, 120, 144, and 168 h for AVB and at 0, 1, 2, 3, and 18 h for CUR. To obtain those of OXB in the absence or presence of β-CD, the invariable samples were certified at 0 and 48 h. For the samples in acetonitrile aqueous solution or methanol aqueous solution at a ratio of 7:3, the time evolution of the UVA1 irradiated solution was examined under similar conditions.

The photostability of the drug under solar light was evaluated using UV–vis spectral measurements. Sample solutions were exposed to solar light outdoors at a sunny location on a latitude of 35.92° N and a longitude of 139.91° E for 4 h, from 12:00 to 16:00, at an average temperature of 284.55 K.

### 2.6 Singular Value Decomposition (SVD) Computation for Spectrometric Data

The *i*th observed spectrum {Φ⃗_*i*_|1 ≤ *i* ≤ *n*} of the sample represents an *m*-dimensional vertical vector measured at a specific wavelength. The wavelength range spans 230–700 nm with an interval of 1 nm, resulting in *m* = 471. Matrix M, defined in eq 5, consists of a horizontal sequence of the obtained spectral vectors (with dimensions *m* × *n* = 471 × 64).
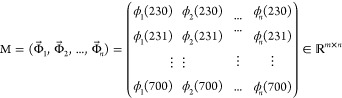
5M and M^*t*^ represent the real and transposed matrixes, respectively. The products M^*t*^M and MM^*t*^ form orthogonal matrixes. The matrixes describing M can be transformed into eq 6.
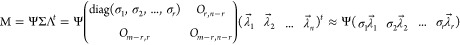
6The matrix ∑ comprises the diagonal elements {σ_*i*_|1 ≤ *i* ≤ *r*} (positive absolute values ordered in descending order representing singular values denoting dispersion). The *i*th column of the orthogonal matrix Λ is the coefficient vector corresponding to the singular value (σ_*i*_) and vector (λ⃗_*i*_) is a specific singular vector. The rows of matrix Ψ are considered basis function vectors; the principal component vector (ω⃗_*i*_) results from the product of λ⃗_*i*_ and the corresponding σ_*i*_.
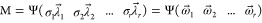
7Matrix Ψ comprises rows that are basis function vectors. We applied SVD to a 471 × 64 spectral data matrix. The dimensionality was determined based on the logarithm of the singular value against the index, establishing the minimum dimensionality required to replicate the vector space of the document spectrum. The value can be almost negligible if the singular value is much smaller than a certain threshold (e.g., several hundredths of the highest singular value). The chosen dimensionality *r* enables the principal components to approximately reproduce the vector space, including the document spectrum as the *j*–*h* vector (*x⃗*_*j*_) composed of the *i*th elements (*x*_*i*,*j*_), as described in eq 8.^53−60^
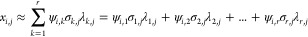
8

### 2.7 Nuclear Magnetic Resonance (NMR) Spectroscopy

^1^H NMR measurements were conducted using a 400 MHz NMR spectrometer (JNM-ECZ 400 S, Japan Electronics Co., Ltd., Tokyo, Japan). Sample solutions were prepared using D_2_O and methanol-*d*_4_ as the protic solvent, acetonitrile-*d*_3_ as the aprotic solvent, and their mixtures, with sample concentrations exceeding 1.5% (w/v). The chemical shifts of the samples were calibrated using the internal tetramethylsilane (TMS) signal as the zero point, whereas the solvent signals in the literature served as reference points. When sodium salts were required, the neutral AVB species in D_2_O were dissolved in equimolar amounts of aqueous NaOH and methanol-*d*_4_, followed by drying under reduced pressure in a rotary evaporator and heating to dryness below *T*_m_ of the sodium salts. The formation of sodium salts was confirmed by measuring *T*_m_ using a differential scanning calorimeter (DSC8230, Rigaku Co., Ltd., Tokyo, Japan).

^1^H–^1^H homonuclear correlation spectroscopy (COSY) involves scanning electromagnetic radiation pulses through hydrogen nuclei, eliciting responses from resonant hydrogen atoms with geminal, vicinal, or long-range coupling. The diagonal signal corresponds to the hydrogen response to the scanned radio waves at a specific frequency, whereas cross peaks that do not align with the diagonal reveal adjacent hydrogens. ^1^H–^1^H homonuclear Overhauser effect spectroscopy (NOESY) identifies signals arising from hydrogen atoms in close spatial proximity, providing through-space correlations via spin–lattice relaxation. For optimal spectral assignment by NOESY, the mixing time should fall between half of *T*_1_ and *T*_1_, with increasing longitudinal relaxation time, enhancing NOESY sensitivity. This can be achieved by selecting a low-viscosity solvent (such as acetone-*d*_6_) and removing the dissolved oxygen from the sample.

^1^H–^13^C heteronuclear multiple quantum coherence (HMQC) measurements detect coupling cross peaks between carbon-13 nuclei adjacent to scanning protons. The experiments were conducted according to a previously described protocol.^22^ When the sample produced confusing signals in the HMQC experiments, ^1^H–^13^C heteronuclear single quantum coherence (HSQC) measurements were used to obtain a higher resolution. ^1^H–^13^C heteronuclear multiple bond coherence (HMBC) measurements identify coupling cross peaks between the carbon-13 nuclei and scanning protons through two or three bonds. This measurement followed the experimental protocol.

### 2.8 NMR Titration for the Tautomeric Species of AVB

In the one-dimensional (1D) ^1^H NMR spectra, the integral intensities correspond to the relative stoichiometry of protons for the species, except for the signals assigned to the stable isotopes of deuterated solvents. We could recognize the aromatic proton signals of AVB, but the corresponding signals sometimes overlapped between its keto-enol and diketo species, depending upon the solvent composition. The signals of the aromatic protons at the *para* positions to the keto/enol substituents were neglected because of the high probability of overlap, in which the chemical shift drifts, Δδ, related to the shielding effect are not noticeably accompanied by the electronic states in the keto/enol substituents.

We chose the signals of the aromatic protons at the *ortho* positions and the aliphatic protons at the *ortho* substituents (*tert*-butyl and methoxy groups) to keto/enol substituents and measured their integral intensities. Using the simultaneous equations built as linear combinations of integral values for the isolated and overlapping signals, we determined the molar ratio of the keto-enol and diketo species.

## 3 Results and Discussion

### 3.1 1-Octanol/Water Partition Coefficients of AVB, OXB, and CUR

**Figure 1:**
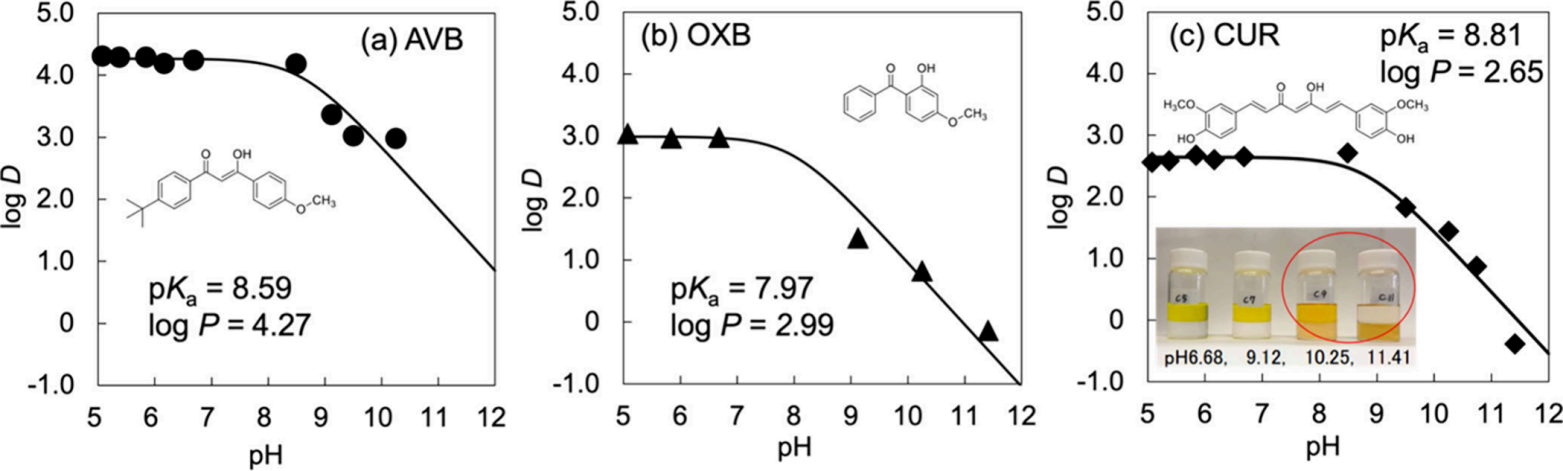
pH profiles of distribution coefficients, log *D*, for (a) AVB, (b) OXB, and (c) CUR. The 1-octanol/water (the modified Britton–Robinson buffers at pH 5–11.5) partition coefficients, log *P*, the acid dissociation constants, p*K*_a_, were optimized using curve fitting approximated to log *D* = (log *P*)/(1 + 10^pH–p*K*_a_^), which is Avdeef’s diagram^41^ derived from the Henderson–Hasselbalch expression for the equilibrium between the protonated and deprotonated species. Curve-fitting procedures employed the Solver module of Microsoft Excel 2016 with the implemented GRG nonlinear option. The approximated curves corresponding to these optimized parameters were drawn, and the p*K*_a_ and log *P* values for AVB, OXB, and CUR were represented. The inset photograph shows the equilibrium CUR partitioned in 1-octanol/water phases at several pH values.

AVB absorbed a unique UVA1 band, whereas OBZ was comparatively verified as an UV filter absorbing UVB (280–320 nm) and UVA2 (320–340 nm) light with a benzophenone moiety (cf. Chart 1).^36^ AVB has a bibenzoylmethane scaffold, whereas CUR was used as an antioxidant reference for diketomethane and keto-enol tautomerization.^61^ We evaluated the hydrophobicity of these samples using the flask-shaking method to determine the partition coefficient between the 1-octanol and aqueous phases,^41−44^ in which the pH of the aqueous phase was regulated between 5.0 and 11.5 with a modified Britton–Robinson universal buffer. The limits of detection for the calibration curves of AVB, OXB, and CUR prepared using reversed-phase HPLC were 0.0055, 0.0323, and 0.0030 mM, respectively, while the limits of quantitation were 0.0142, 0.0881, and 0.0112 mM, respectively. Figure 1 shows the pH profiles of the apparent partition coefficients (distribution coefficients), and log *D* for AVB, OXB, and CUR. Curve fitting for Avdeef’s log *D*–pH diagram for acidic compounds was used to estimate the p*K*_a_ and log *P* values for the neutral form.^41^ The reported p*K*_a_ values of AVB and OXB are 9.7 and 7.6, and the log *P* values of AVB and OXB are 4.51 and 3.79.^62^ CUR has three p*K*_a_ values of 7.8, 8.5, and 9.0, corresponding to the keto-enol, first phenolic OH, and second phenolic OH, respectively.^63^ The updated p*K*_a_ values of CUR were 7.56, 8.72, and 10.17,^64^ and its log *P* was 3.2.^65^ Our obtained p*K*_a_ and log *P* values were consistent with published values, but the order of log *P* values was AVB (4.27) > OXB (2.99) > CUR (2.65). Curcumin contains a keto-enol site and two phenolic hydroxyl groups. The acid dissociation constants of dissociable groups with the same structure change consecutively. In other words, the acid dissociation constant of one group changes depending upon whether or not the other group has dissociated. Cantor and Schimmel demonstrated that the acid dissociation constant of the terminal group in oligopeptides composed of alanine changes with peptide chain length.^66^ Therefore, it is challenging to distinguish the apparent p*K*_a_ values derived from the two phenolic hydroxyl groups. In Figure 1c, only one p*K*_a_ was calculated; however, the plot at pH 11.5 showed lower values compared to Avdeef’s log–pH plot. This suggests that the dissociation of the second phenolic hydroxyl group is occurring. This confirmed the presence of a few ionized forms of AVB, OXB, and CUR at pH 6.8.

### 3.2 Phase-Solubility Diagrams of AVB, OXB, and CUR with CDs

The CDs are a macrocyclic hexamer (α-CD), heptamer (β-CD), and octamer (γ-CD), that consist of glucopyranosides linked by α1–4-glycoside bonds.^46,67^ Consisting of a cavity (with a diameter of 0.60–0.65 nm) appropriate to include various phenyl portions, β-CD has been widely used for the mechanochemical syntheses of pharmaceutics.^67^ Because its use is limited owing to its notable low aqueous solubility (16.3 mmol/L) and nephrotoxicity suspicion, especially in parenteral drug delivery,^67^ alternatively, the partially 2-hydroxypropylated derivative of β-CD (HP-β-CD) was developed, which is more highly soluble (more than 400 mmol/L) and can be chemically degraded and photodegraded.^65^ The derivative is considered to have higher solubility and greater nontoxicity at low to moderate oral and intravenous doses.^67^ Additionally, the derivative has generally been verified as safe and is administered parenterally to animals and humans.^68^

**Figure 2:**
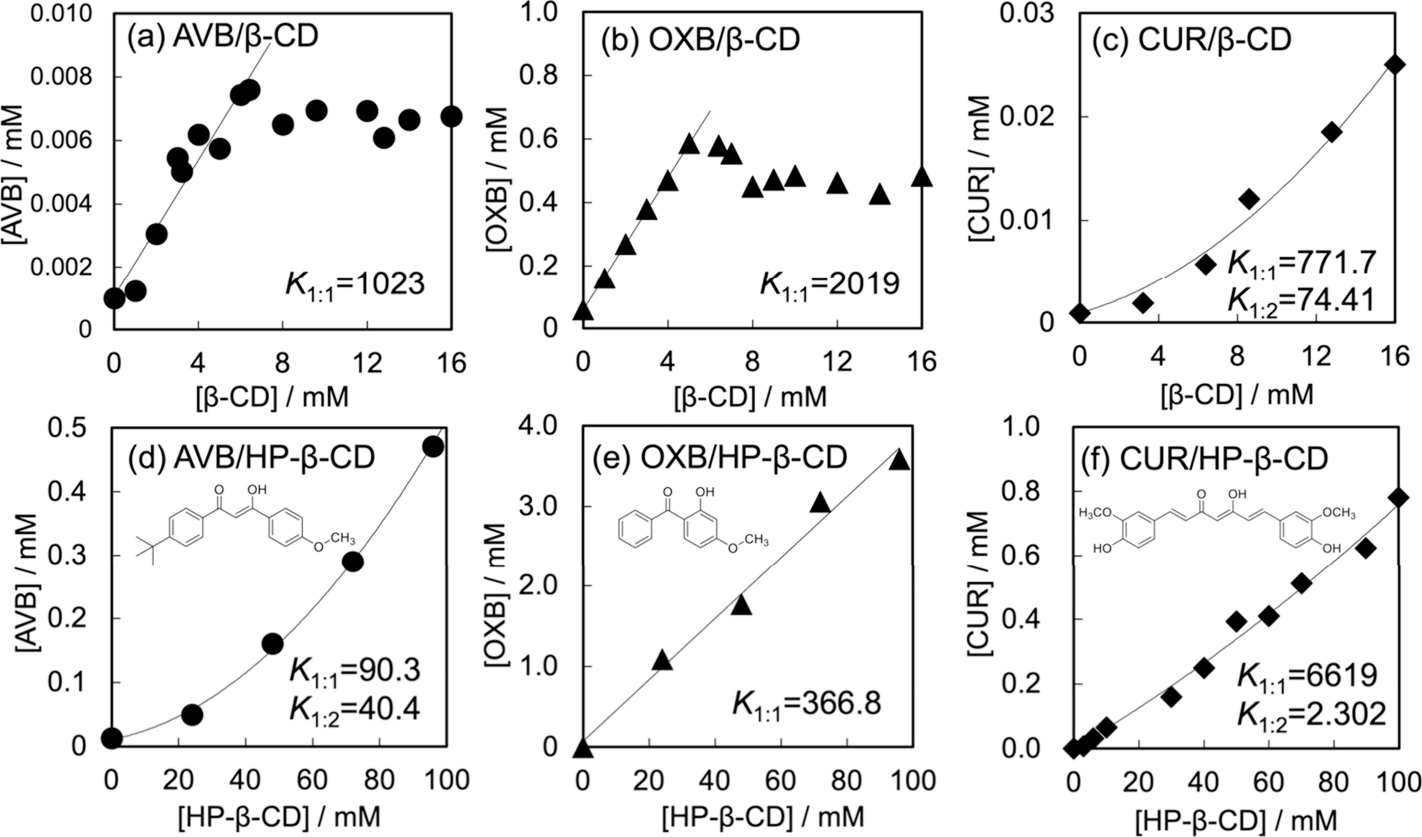
Phase-solubility diagrams of (a) AVB, (d) OXB, and (c) CUR with β-CD and (d) AVB, (e) OXB, and (f) CUR with HP-β-CD in 25 mM P_i_/NaOH buffer at pH 6.8. The regression lines for panels a, b, and e provided the determination coefficients, 0.9223, 0.9994, and 0.9872. The parabolic curves for panels c, d, and f brought the determination coefficients, 0.9946, 0.9995, and 0.9901.

Figure 2 shows the phase-solubility diagrams of AVB, OXB, and CUR to β-CD or HP-β-CD. In panels a and b of Figure 2, the apparent solubility to β-CD of AVB and OXB increases linearly, but plateaus appear at concentrations greater than 5 or 6 mM of β-CD. We attempted to explain their profiles using Langmuir adsorption isotherms and Hanes–Woolf plots, which proved unsuitable. Vishwakarma et al.^69^ reported the Benesi–Hildebrand analyses of the phase-solubility diagram for ABZ and β-CD, and concluded that linear or parabolic relationships prevailed; that is, the stoichiometry of AVB and β-CD was 1:1 or 1:2, when the researchers examined less than 1 mM β-CD. Given that our results indicated a linear correlation, we confirmed the equimolar complex of AVB and β-CD in the 0–6 mM range. Precipitation was observed at the higher concentration of β-CD, which might have suspended the AVB/β-CD or OXB/β-CD inclusion complex. Sbora et al.^70^ determined the molar ratio of AVB with β-CD and HP-β-CD to be 1:2 in the solid lyophilized inclusion. The theoretical study of Vishwakarma et al.^69^ supported the probability of the 1:2 inclusion complex of AVB/β-CD.

The pattern shown in Figure 2b is consistent with that reported by Sarveiya et al.^71^ Although the phase-solubility curves of AVB and OXB for β-CD appeared to correspond to the B_S_-type in the conventional catalog of Higuchi and Connors,^45−48^ they differed from the B_S_-pattern; the appearance of overhangs above the plateau region in the higher β-CD concentration, confirmed the results obtained by Simeoni et al.^72^ Assuming the suspended AVB/β-CD inclusion complex would transform from the equimolar form to the double-side-capped complex (with a stoichiometry of 1:2) at the higher β-CD concentration, as mentioned above, the gap between the highest solubility on the linear correlation (i.e., the formation of the equimolar complex) and plateau level (i.e., the double-side-capped complex or more coagulated particles) could be acceptable as an indication of the difference in the solubility (dispersion concentration) of the different forms. We observed that precipitation probably corresponded to this gap.

**Figure 3:**
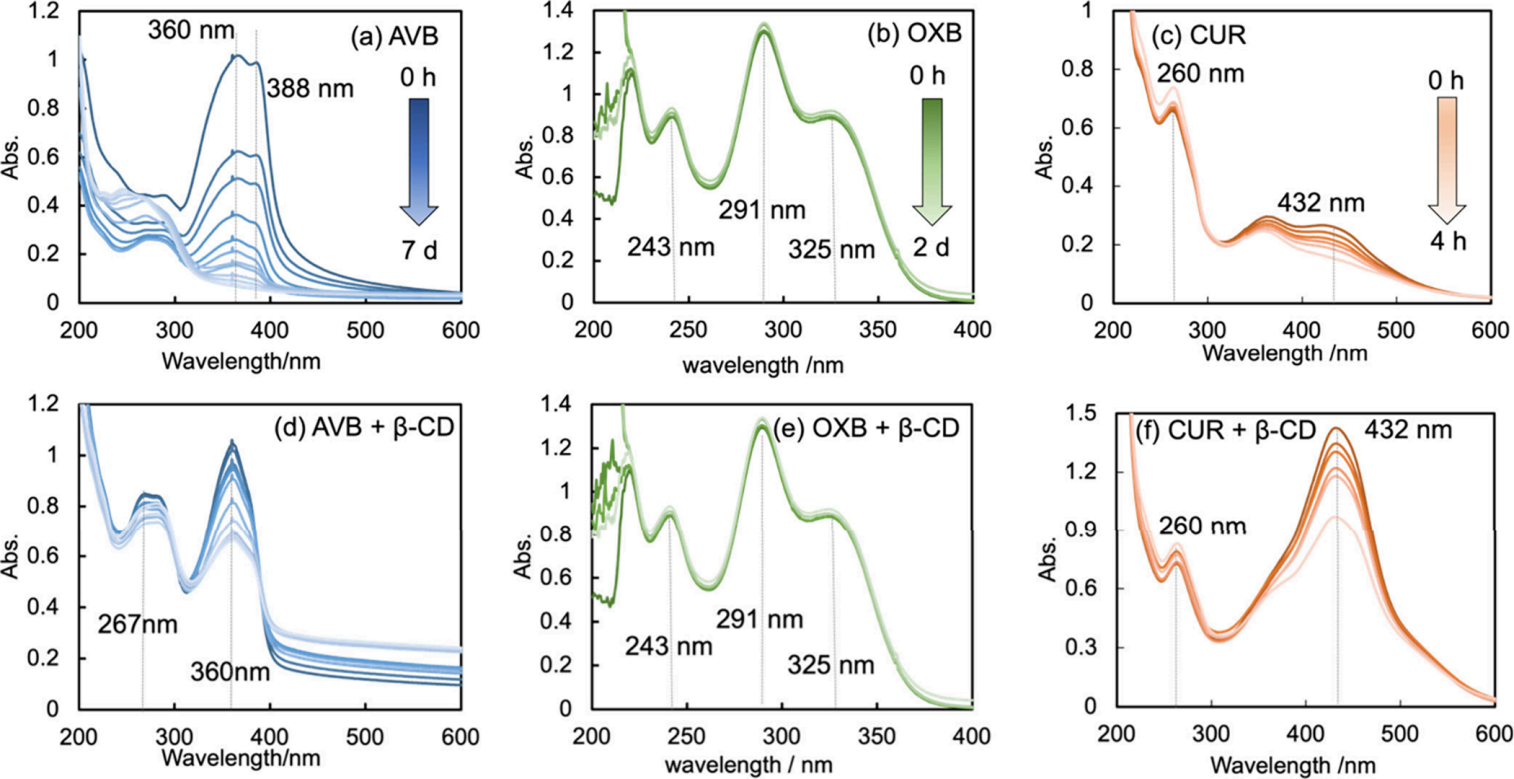
UV–vis spectra of 50 μM (a) AVB, (b) OXB, and (c) CUR irradiated with the UVA1 (365 nm) lamp in 25 mM Tris–HCl (pH 7.4). The spectra of panels d–f correspond to these drugs irradiated with the UVA1 lamp in 8 mM β-CD.

Figure 2e indicates that the apparent solubility of OXB increases linearly in the diagram for 0–100 mM HP-β-CD (*r*^2^ = 0.9872). Corresponding to the A_L_-type described by Higuchi and Conners,^45^ this phenomenon indicates the formation of the OXB/HP-β-CD equimolar complex. Despite resembling the results obtained for OXB/HP-β-CD (0–60 mM) by Sarveiya et al.,^71^ a notable difference is evident; the slope obtained by Sarveiya et al.^71^ overlapped the results for OXB/HP-β-CD approximately 4 times in Figure 2e. This difference was probably caused by differences in the experimental conditions (solute concentration, pH, or temperature). Simeoni et al.^72^ reported a lower OXB/HP-β-CD diagram stability constant. A generality of the A_L_-type correlations observed in the OXB inclusion complexes with other cyclodextrins: HP-α-CD, HP-γ-CD, and sulfobutyl ether (SBE)-β-CD was evident. Because the UV–vis spectrum of OXB remained almost unchanged in the absence and presence of 4 mM β-CD before UVA1 irradiation (Figure 3), the electronic structure of OXB was sustained in the solution or the equimolar inclusion complex.

Figure 2d shows the parabolic curve obtained for AVB/HP-β-CD. The physicochemical properties, photodegradation, and chemical stability of many inclusion/encapsulated complexes of AVB with HP-β-CD have been studied.^36−38^ As Yang et al.^73^ and Yuan et al.^74^ published parabolic curves similar to those of the AVB/HP-β-CD phase-solubility diagrams obtained in this study, the AVB complex can be assumed to be double-side-capped with two HP-β-CDs at both benzoyl sides of AVB. Using traditional regression analysis with the 2-order equation, we obtained the stability constants for an equimolar and 1:2-ratio complex of *K*_1:1_ = 90.3 L/mol and *K*_1:2_ = 40.4 L^2^/mol^2^ (*r*^2^ = 0.9995). The stability constants indicate that the affinity of AVB for β-CD (*K*_1:1_ = 1023 L/mol, *r*^2^ = 0.9223) is approximately 10 times greater than that for HP-β-CD as the equimolar complex.

Panels c and f of Figure 2 illustrate the Higuchi and Conners A_P_-type curves^45^ obtained for the CUR/β-CD and CUR/HP-β-CD complexes. This suggests that the double-side-capped CUR complex comprises cyclodextrins with equivalent feruloyl (i.e., 4-hydroxy-3-methoxycinnamoyl) moieties.^75,76^ Arya and Raghav^77^ justified this 1:2 stoichiometric ratio for CUR/β-CD. Although Karpkird et al.^78^ appropriated an A_L_-type line (*r*^2^ = 0.9464), their plots appear to follow a downward convex curve. Jahed et al.^79^ proposed an A_L_-type relationship between CUR solubility and the β-CD concentrations. However, the diagram reported by Jahed et al.^79^ included a result for 20 mM on the abscissa, which was excess to the β-CD solubility. Reducing the plot to a maximum of 16.3 mM was expected to yield a gradual parabolic curve.

Ghanghoria et al.^80^ indicated that the phase-solubility diagram was of the B_S_-type. However, the researchers did not report the total amount of CUR in their screw-capped vials, and we speculated that CUR solubility was not greater than 0.016 mM. Mashaqbeh et al.^81^ also reported an A_N_-type diagram (saturated at most with 0.00012 mM CUR) that was examined under similar circumstances. Interpretations of the physicochemical and quantitative properties of dietary ingredients and CDs in literature require reasonable agreement. Although we considered the above reports did not essentially contradict our parabolic curves, we failed to rationalize that CUR/β-CD and CUR/HP-β-CD provide the A_L_-type phase-solubility diagrams reported by Jantarat et al.^82^ On the basis of our results, the diagrams for CUR/β-CD and CUR/HP-β-CD should be parabolic. The calculated *K*_1:1_ values for CUR with β-CD was 771.7 M^–1^. This *K*_1:1_ value is in the same order of magnitude as described by Mashaqbef et al.^81^ (487.3 M^–1^) and Chen et al.^83^ (198 M^–1^). In the same way, the *K*_1:1_ value of the CUR/HP-β-CD is 6619 M^–1^, it is in the same order of magnitude as described by Celebioglu and Uyar^84^ (3073 M^–1^) and Li et al.^85^ (2941 M^–1^). It was confirmed that the singly occupied complex of CUR with cyclodextrin is predominant, and the *K*_1:1_ value for CUR/HP-β-CD (*r*^2^ = 0.9901) is 8.6 times higher than that for CUR/β-CD (*r*^2^ = 0.9946).

In conclusion, the apparent solubility of AVB, OXB, and CUR increased in a linear or parabolic manner depending upon the cyclodextrin concentration up to 4 mM. Furthermore, the stability constants for the equimolar complexes of AVB, OXB, and CUR to β-CD were more significant than those to HP-β-CD. Although HP-β-CD was used in many studies to obtain the AVB or OXB inclusion complexes,^70−74,82^ we confirmed that the efficacy of forming the β-CD inclusion was higher than that of forming the HP-β-CD inclusion. The stability constants were independent of the hydrophobicity of AVB, OXB, and CUR. Further study focused on the effects of this CD because β-CD efficiently improved the AVB aqueous solubility and held no structural diversity.

### 3.3 UV–Vis Absorption Spectral Change of AVB with/without β-CD

Figure 3 shows the UVA1 photodegradation of 50 μM AVB, OXB, and CUR in the absence or presence of 4 mM β-CD. Absorption peaks of AVB in the aqueous phase were observed at 360 and 388 nm. According to the theoretical study by Sahoo et al.,^86^ the chelated (intramolecular cyclic hydrogen bonding) keto-enol forms of *p*-*tert*-butylbenzoyl-*p*-methoxyphenyl–enol (χ1, form A) and *p*-methoxylbenzoyl-*p*-*tert*-butylphenyl–enol (χ2, form B) correspond to the higher and lower wavelength peaks, respectively. In various solvent studies performed by Mturi and Martincigh,^87^ the maximum peaks (360 nm in the aqueous phase) were found at 365 nm in dimethyl sulfoxide (DMSO), 358 nm in methanol, 355 nm in ethyl acetate, and 350 nm in cyclohexane. As the chelated form is dominant in nonpolar solvents, the 360 and 388 nm peaks could be considered as corresponding to the chelated and nonchelated forms. In the presence of β-CD, the AVB spectrum lacks a shoulder at 388 nm. Assuming AVB intrudes into the hydrophobic internal cavity of β-CD, hydrogen bonding becomes dominant. This suggests the 388 nm peak corresponds to the nonchelated form. In a hydrophobic environment, the diketo form of AVB increases to enhance the 267 nm peak.

**Figure 4:**
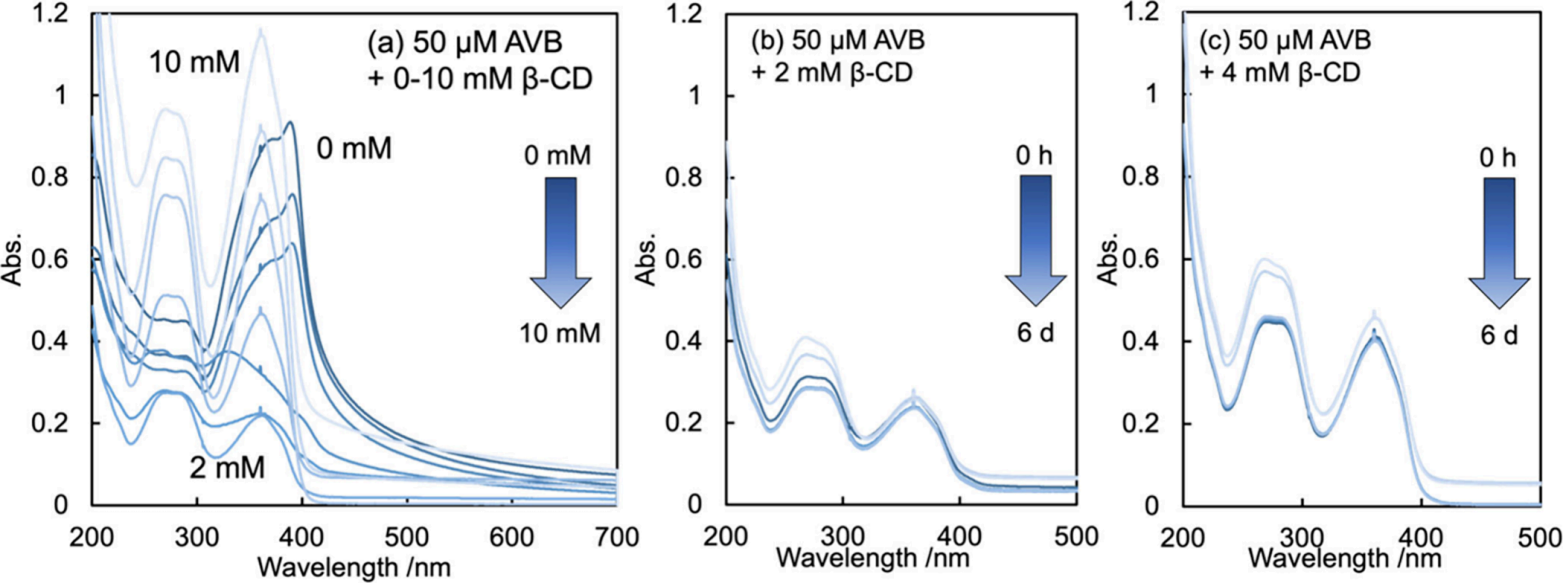
(a) UV–vis spectral titration for 50 μM AVB with 0–10 mM β-CD. In 25 mM P_i_ buffer (pH 6.5). The spectra of AVB were represented with gradually diluted curves depending upon the concentration of β-CD. They peaked at 388 nm, and spectra gradually morphed and descended with 0, 1/40, 1/20, 1/10, 1, and 2 mM β-CD. Furthermore, twin peaks at 274 and 365 nm expanded with 2, 4, 6, 8, and 10 mM β-CD. The signal at 388 nm would be assigned to the keto-enol species in the aqueous phase, while the signals at 274 and 365 nm would be to the diketo and chelated keto-enol species in β-CD’s hydrophobic internal cavity, respectively. UVA1 irradiation insignificantly degraded AVB with (b) 2 mM and (c) 4 mM β-CD for 0–6 days.

Figure 4a shows the titration results obtained for 50 μM AVB with 0–10 mM β-CD. The 360 and 388 nm peaks of AVB were observed in the absence of β-CD and gradually increased depending upon the β-CD concentration which ranged between 0 and 2 mM. However, when the β-CD concentration increased to more than 2 mM, the 388 nm peak disappeared, but the 267 and 360 nm peaks increased gradually. Vishwakarma et al.^69^ reported that the spectra of 10 μM AVB decreased for 0–1 mM β-CD and increased for 1–10 mM β-CD, indicating that the absorption change switched from decrease to increase at a threshold of 80–100 times the β-CD concentration to AVB. According to the researchers, the peak decrease at 360 and 388 nm for β-CD concentrations less than this threshold is induced owing to a polarity effect on the main absorption band of a π–π* nature in AVB. The switch to the increment of the 360 nm peak at a higher β-CD concentration than the threshold indicates the possibility of multiple host–guest complexation. This diversity is consistent with the possibility of the divalent binding of AVB to β-CDs at concentrations greater than 6 mM in the phase-solubility diagram (Figure 2a).

**Figure 5:**
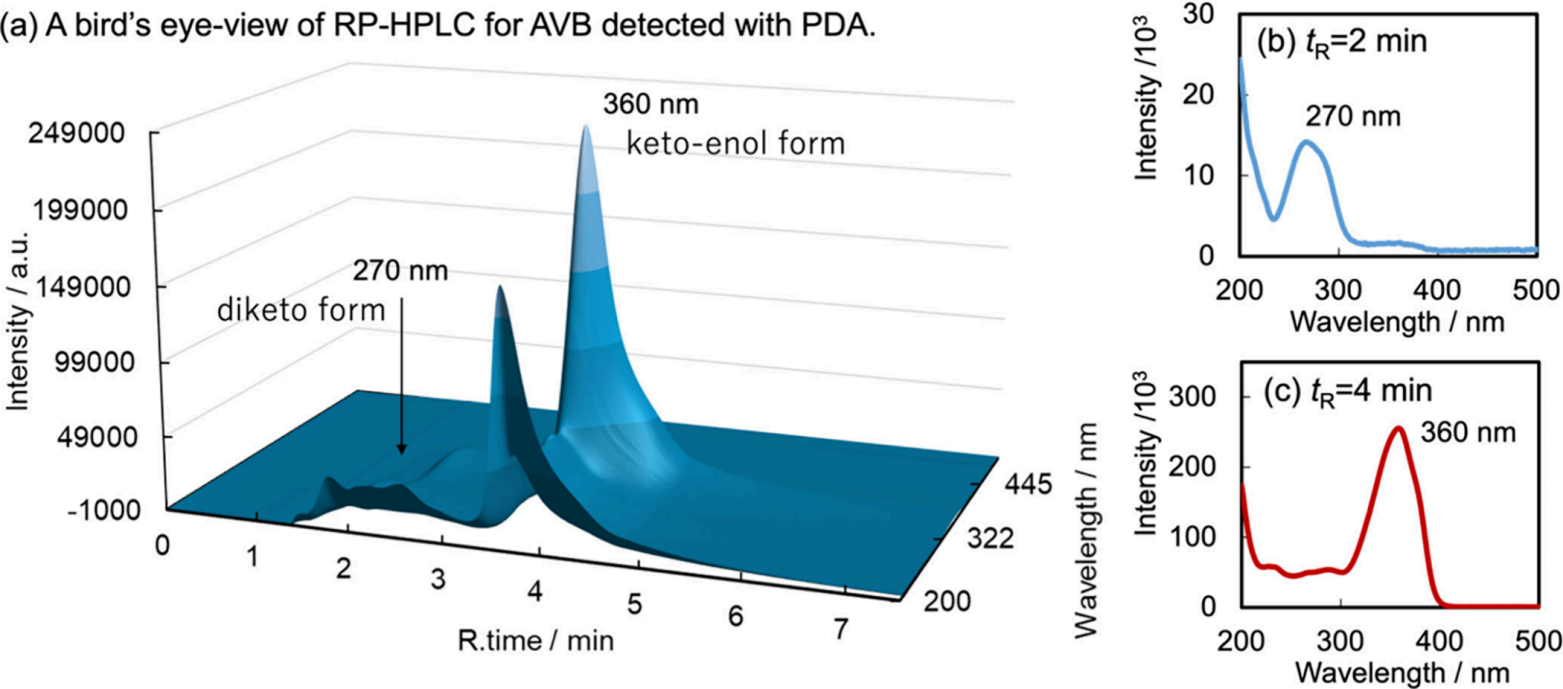
(a) Reversed-phase HPLC chromatogram of intact AVB. Stationary and mobile phases were the ODS column and the Pi buffer (pH 2.5) in H_2_O/acetonitrile = 1:1, respectively. Signals were observed with the photodiode array (PDA) at 200–600 nm wavelength. Panels b and c represented the cross sections of spectra at the retention times *t*_R_ of 2 and 4 min, respectively. The keto-enol form is planar and absorbs in the UVA1 range (340–400 nm), while the diketo tautomer absorbs in the UVC range (200–280 nm)^81^.

Figure 5 shows the reversed-phase HPLC chromatogram of AVB that was obtained using an isochoric acetonitrile/water solvent as the mobile phase. A component with a 270 nm peak at a retention time of approximately 2 min and a 360 nm peak at 4 min is present. The mobile phase easily eluted the diketo form owing to its hydrogen-bonding acceptors and structural flexibility. In contrast, the stationary phase could retain the chelated keto-enol form because of the shielded acceptor and expanded planar structure.

The spectra of OXB and the OXB/β-CD shown in Figure 3, exhibit no noticeable difference in the peak heights at 243, 291, and 325 nm. The spectral peak at 432 nm of the CUR/β-CD complex is narrower than that of the neat CUR because CUR would intrude into the hydrophobic internal cavity of β-CD to locate in an apolar atmosphere and restrict its fluctuation. In contrast, the OXB spectra that exhibit no difference indicated that the complexation of OXB into β-CD afforded no changes. Al-Rawashdeh et al.^88^ confirmed little change in the NMR chemical shifts of OXB and its complexes. In their scheme, based on theoretical computations, the benzophenone moiety intrudes into the hydrophobic internal cavity of β-CD, and its keto-phenol, in which the hydrogen acceptor site interacts with one of the C6-OH groups in β-CD, is held at the interface between the internal cavity and external aqueous phase.

### 3.4 Photodegradation of AVB, OXB, and CUR in Aqueous Solution

As shown in Figure 3 and panels b and c of Figure 4, the UVA1 lamp irradiated at 365 nm. In the absence of β-CD, AVB gradually photodegraded during UVA1 irradiation for a week and exhibited a hypochromic effect on the absorbance at λ = 267, 360, and 388 nm; the byproducts increased as indicated by the peak at 250 nm. In the presence of β-CD, the 360 nm peak decreased slowly and partially, and the 267 nm peak decreased more slowly than the 360 nm and partially. The increase in the intensity of the peak or shoulder at 250 nm was insignificant, suggesting the protection from AVB photodegradation. Panels b and c of Figure 4 demonstrate UVA1 irradiation insignificantly degraded AVB upon adding 2 mM and 4 mM β-CD. β-CD with a molarity 80–100 times that of AVB induced the hypochromic effect on the absorbance of AVB and prevented its UVA1 photodegradation.

Figure S1 shows the RP-HPLC patterns measured at λ = 360 nm (panels a and c) and 270 nm (panels b and d). Without β-CD, the signals at the retention time *t*_R_ of 2.2 min observed at λ = 360 and 270 nm decreased during 2-d irradiation. Thereafter, the signals at *t*_R_ = 1 and 1.4 min observed at λ = 270 nm increased from 2 days of irradiation onward, suggesting AVB was photodegraded during this irradiation. With β-CD, the signal at *t*_R_ = 2.2 min observed at λ = 360 nm decreased slightly during 7 days of irradiation. The signals at *t*_R_ = 2.2 and 1.2 min observed at λ = 270 nm remained. The signal at 1 min increased marginally, indicating that AVB in its complex with β-CD was partially photodegraded. Although the signal heights could not be compared between the 360 and 270 nm signals, the 360 nm signal decreased gradually, but the 270 nm signal increased. The keto-enol species encapsulated in the β-CD complex transformed into the diketo species, certifying photostabilization under the 365 nm lamp. The keto-enol species encapsulated in the β-CD complex transformed into the diketo species as if AVB intended to escape photodegradation under UV irradiation. This metaphor reflects a crucial concept of this study.

Because OXB absorbs light in the UVB (280–320 nm) and UVA2 (320–340 nm) regions, the 365 nm lamp could not presumably affect the OXB and the OXB/β-CD complex. Panels b and e of Figure 3 show the maintenance of the OXB spectrum for 2 days. CUR exhibits an absorption spectrum with a peak at 432 nm, which increased by the formation of an inclusion complex with β-CD (panels c and f of Figure 3). When exposed to UV light for 4 h, the CUR solution containing β-CD displayed disruption of 31.90%, compared to that (40.95%) by the CUR solution without β-CD. These results indicate that the formation of the inclusion complex between CUR and β-CD helps suppress the photodegradation of CUR. Many scholars have complexed curcumin with CDs to protect the molecule from photodegradation.^89^ However, marked differences are evident in the photostabilization effects of various cyclodextrins.^90^ Complex formation has a destabilizing effect rather than a photostabilizing effect on the photodegradation process, and an increase has been observed in the degradation rate.^89^ In organic solvents, CUR decomposes upon exposure to light. Whether the hydrophobic internal cavity of β-CD renders CUR photostabilizing depends upon the situation.

Figure S2 shows the RP-HPLC chromatogram of CUR containing these three components. The mobile phase comprised 40% acetonitrile in phosphate buffer (pH 2.5). The spectra of the components exhibit single peaks at 430, 420, and 415 nm. In the aqueous phase, the spectra of CUR and the CUR/β-CD complex peaked at 430 nm and shouldered at 360 nm; the shoulder did not correspond to the components of CUR, thereby suggesting the diketo tautomer of CUR. The 360 nm peak of the chelated keto-enol tautomer of CUR could become narrower owing to the formation of the inclusion complex with β-CD.

Figure S3 shows the changes in UV–vis spectra of AVB, OXB, and CUR in the presence and absence of β-CD under solar light. The evaluation of drug stability under solar light was conducted by modifying a previously reported method.^91^ For AVB, no significant changes in stability were observed under solar light exposure, regardless of the presence or absence of β-CD, compared to UVA1 irradiation. In the case of OXB, the change in absorption peaks due to solar light exposure was more significant than that observed under UVA1 irradiation. Because OXB is reported to be unstable to UVB (280–320 nm),^92^ it is presumed that the slight UVB present in solar light accelerated the degradation of OXB. For CUR, significant changes in absorption peaks were observed under solar light in the presence of β-CD. These changes were not observed under UVA1 irradiation, suggesting that UVB and visible light present in solar light may affect the stability of CUR. Additionally, in AVB and CUR solutions containing β-CD, precipitation was observed under solar light exposure, resulting in an elevated baseline.

Conclusively, although β-CD encapsulation can occasionally enhance and, in contrast, suppress the stability of the guest,^27,51^ β-CD canceled the photodegradation of AVB.

### 3.5 Photodegradation of AVB, OXB, and CUR in 50% Acetonitrile Solution

In section 3.3, we discussed the effect of β-CD encapsulation on the spectrum of AVB, which indicated that the contribution of UVA1 irradiation on the AVB/β-CD complex cannot directly compare with that on neat AVB. Figure 5 indicates the multiple retention times at λ = 270 nm for the diketo forms corresponding to their rotamers and the single retention time at λ = 360 nm for the chelated keto-enol form. Therefore, the mobile phase (acetonitrile/H_2_O = 1:1) avoids the structural diversity of the keto-enol species in an aqueous solution. Hanson et al.^33^ reported that SDS micellar encapsulation transforms the AVB spectrum to a level similar to that of methanol. The researchers discovered that AVB photodegradation due to solar-mimicry light irradiation progressed in water or cyclohexane but was completely inhibited in dry methanol or 20 mM SDS aqueous solution (AVB to be quarantined from the water phase). Our preliminary experiments allowed the aqueous solvent containing isochoric acetonitrile to mimic the hydrophobic atmosphere in the internal cavity of β-CD.

**Figure 6:**
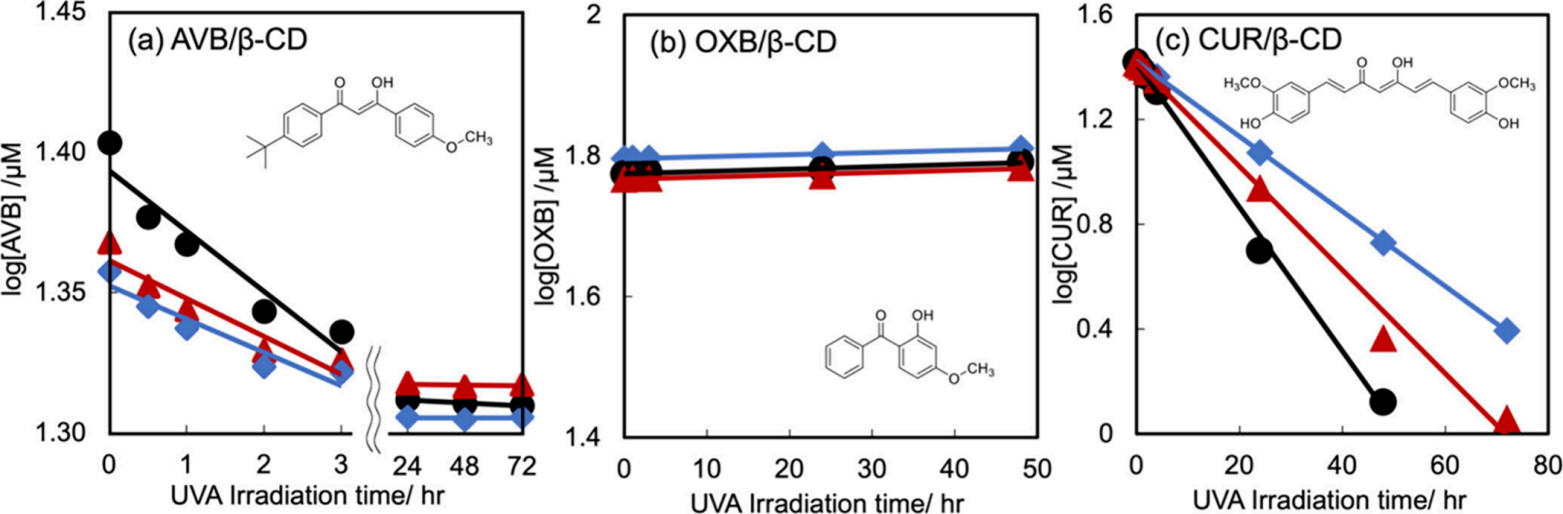
Time evolution of the degradation of (a) 25 μM AVB, (b) 50 μM OXB, and (c) 25 μM CUR in the absence (black circles) and presence (equimolar, Bordeaux triangles; 4 mM, indigo rhombuses) of β-CD in isochoric acetonitrile solvent under the UVA1 irradiation (365 nm). AVB, OXB, and CUR signals and their calibration lines were determined wavelengths at 360, 290, and 430 nm, respectively. The calibration lines provided *r*^2^ = 0.999 and 3σ = 0.022. The obtained diagrams of the logarithm of AVB concentration to the reaction time seemed to have downward convex curves rather than first-order linear functions. Figures S4–S6 represented the corresponding observed spectra.

In this study, we examined the effect of UVA1 irradiation on AVB, OXB, and CUR in the absence or presence of β-CD in an isochoric acetonitrile/water solvent, as shown in Figures 4, 5, and 6, respectively. In this solvent, the AVB photodegradation products were minimal. Adding equimolar or 4 mM β-CD did little to modify the observed 25 μM AVB, OXB, and CUR spectra of this solvent. UVA1 irradiation of AVB involved a 360 nm peak reduction and 270 nm peak increase. These spectral drifts were slightly attenuated in the presence of β-CD. We determined the AVB concentration based on the calibration line of the absorbance at λ = 360 nm of the AVB standard. Figure 6a shows the time course of the AVB concentration under UVA1 irradiation. Equimolar amounts and 4 mM β-CD reduced the photodegradation velocity of AVB. However, the obtained diagrams of the logarithm of the reactant concentration versus reaction time appeared to have downward convex curves instead of first-order linear functions. This implies that the photodegradation of AVB followed a higher-dimensional or composite reaction. Furthermore, as their intercepts decreased depending upon the β-CD concentration, the opposite equilibrium between the keto-enol (360 nm peak) and diketo (270 nm peak) species might compete with the photodegradation. In other words, the keto-enol species in this solvent were interconverted into diketo species, as if AVB intended to escape photodegradation under UVA1 irradiation.

UVA1 irradiation did not significantly influence the spectra of OXB in the isochoric acetonitrile/water solvent. Figure 6b indicates that the OXB concentration derived from the 290 nm absorbance was marginally reduced by UVA1 irradiation for up to 50 h, as expected. As shown in Figure S7, a longer duration caused peaks intensities in the CUR spectra to decrease. Adding β-CD delayed the CUR decrements under irradiation. Figure 6c shows the time course of the CUR concentration observed as the absorbance at λ = 430 nm and first-order reaction rate of the photodegradation of CUR, which decreased upon adding the equimolar and 4 mM β-CD. Mangolim et al.^75^ explained that the primary degradation product is the cyclization of curcumin due to the loss of two hydrogen atoms from the molecule, which is also derived from vanillin, vanillic acid, ferulic aldehyde, ferulic acid, and 4-vinyl guaiacol.^63^ These tendencies do not contradict our results for the first-order degradation of CUR.

We conclude that the photodegradation kinetics of AVB involve a composite reaction process, such as keto-enol and diketo tautomerization.

### 3.6 ^1^H NMR Titration of the Keto-Enol and Diketo Forms of AVB

**Figure 7:**
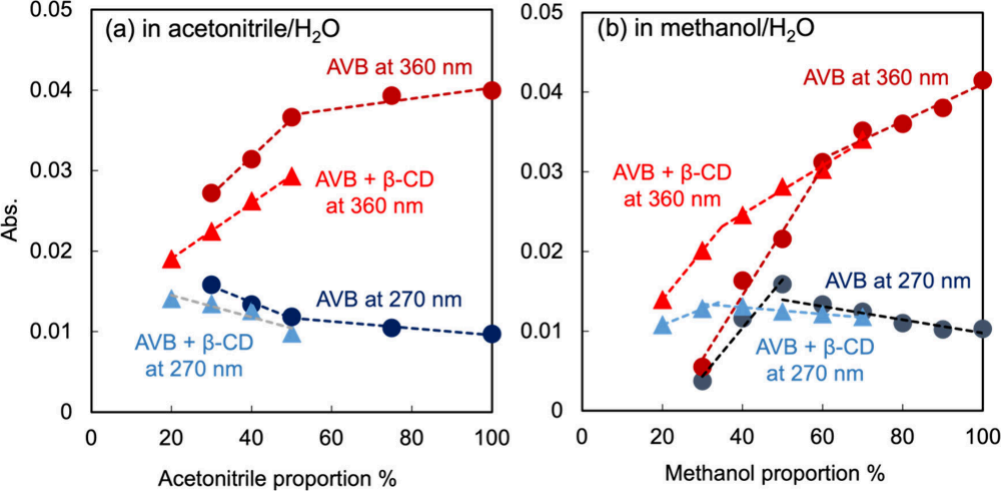
Absorbance of AVB at 270 and 360 nm in a solvent with various proportions of (a) acetonitrile/H_2_O and (b) methanol/H_2_O. It represented intact ABZ with circles and the equimolar mixture of AVB and β-CD with triangles. Supposing the solvent molecules are homogeneous volume spheres, the geometrical kissing number in three dimensions is 12. It indicates a hexagonal close-packed lattice where a central sphere contacts 3–4 surrounding atoms. Hence, if the solvent component contains a higher volume ratio than 66–75%, the surroundings of a solute would be almost filled with these solvent molecules. As acetonitrile and methanol molecules are distorted, we considered that this threshold proportion would be attenuated. That could be why the bending points are at about 60% (v/v)^24^.

UVA1 irradiation induces the photodegradation of AVB in an isochoric acetonitrile/water solvent. With the 360 nm peak height decreasing along a downward convex curve to the cumulative UV-absorbing energy, the 270 nm peak gradually increased. This indicates that the chelated keto-enol species absorbed UVA1 light and was subsequently photodegraded or partially converted to the diketo species. Figure 7 shows the absorbance of AVB at λ = 270 and 360 nm in a solvent with various proportions of acetonitrile/H_2_O and methanol/H_2_O. Although increasing the 360 nm absorbance induces a reduction in the 270 nm absorbance, the absorbances measured at different wavelengths cannot be quantitatively compared because of the different molar adsorption coefficients. The molar ratio between the coexisting keto-enol and diketo species was used to calibrate the absorbance to the molarity.

In the ^1^H NMR spectrum, the signal integrals of the assigned species provided a molar ratio. Figure S8 shows the ^1^H NMR spectrum of AVB dissolved in methanol-*d*_4_/D_2_O = 7:3 solvent. The signals at δ = 8.1205 ppm (*J* = 9.15 Hz) of the aromatic *ortho*-protons in the *p*-*tert*-butyl benzoyl moiety of the keto-enol species were used as an internal standard with an integral of 2. Their corresponding proton signals at the obvious 8.0782 ppm and hidden 8.0553 ppm (predicted by the *J* value) of the diketone species were partially imposed onto the signals **b** at δ = 8.0530 and 8.0313 ppm (central δ = 8.0422 ppm and *J* = 8.68 Hz) of the aromatic protons in the *p*-methoxyphenyl moiety with an integral intensity of 2.10. The *meta*-proton signals **c** at δ = 7.662 ppm (*J* = 8.24 Hz) of the keto-enol species had an integral intensity of 2.61, indicating contamination by the corresponding proton signals of the diketo species, where their doublet signals at the hidden 7.6640 ppm and obvious 7.6411 ppm partially overlapped onto the doublet signals at δ = 7.6620 and 7.6514 ppm for the keto-enol species. The methoxy-proton signals at δ = 3.9788 and 3.9700 ppm and the *tert*-butyl proton signals **f** at δ = 1.4252 and 1.4057 ppm were separated between species. Kumari et al.^93^ showed the singlet signal of the geminal protons in the dibenzoyl methane at δ = 4.7 ppm for AVB in DMSO-*d*_6_. However, the signal for AVB in the D_2_O mixed solvent appeared to be superimposed on the signal of water (and methanol–OH) in the spectra obtained in this study.^93^

By building simultaneous equations from these integral relationships, we resolved the molar ratio of the keto-enol and diketo species in 70% methanol to obtain 84.7 and 15.3%, respectively. Figure S9 shows molar ratios of 90.7 and 9.3% in the isochoric acetonitrile/water solvent. Figure S10 shows that the absence or presence of β-CD did not affect the chemical shift of AVB. Figure S11 summarizes the ^1^H NMR titrations for the keto-enol/diketo molar ratio of AVB in the acetonitrile-*d*_3_/D_2_O solvent with various proportions and methanol-*d*_4_/D_2_O = 7:3 solvent. The diketo population in 40% acetonitrile-*d*_3_ is 1:5, whereas that in 100% acetonitrile-*d*_3_ is 1:50. The diketo form was more favored in a protic solvent (i.e., D_2_O) than the keto-enol form, in which hydrogen bonds formed between the keto and enol moieties. The chelated keto-enol form (intermolecular cyclic hydrogen bonding) loses its pair of hydrogen bond donors and acceptors.

The keto-enol population decreased, the diketo population increased, and their absolute gradients became gentle, depending upon the proportion of acetonitrile in the solvent mixture. Assuming that the acetonitrile molecule is a sphere arranged in a hexagonal close-packed lattice, the critical probability of forming the internal linkage of the heterogeneous sphere was estimated at 1/3 in the three-dimensional percolation model.^18,24^ Hence, the protic solvent (i.e., water), which occupies 33% of the volume of the acetonitrile solvent, can build a hydrogen bond network. A solute dissolved in 66% or less aqueous acetonitrile can be linked through a hydrogen bond network in such a rough approximation. Therefore, more than 66% of the acetonitrile aqueous solvent has a similar nonpolarity, presumably because the molar ratios of the keto-enol and diketo species provided a saturation of more than 66% acetonitrile-*d*_3_.

### 3.7 Molar Absorbance Coefficient of 270 and 360 nm Peaks

Figure S12 shows that the spectral augmentation is proportional to the amount of AVB in the methanol/H_2_O = 7:3 solvent. From NMR titration, the diketo and enol forms were expected to be 15.3 and 84.7%, respectively. Therefore, the abscissae of the regression lines were condensed to the species contents, leading to the molar absorption coefficients (i.e., absorbance gradients to the molarity) for the diketo and keto-enol forms being approximately 8.1 × 10^7^ and 4.3 × 10^7^ L/mol. Figure S13 confirms that the molar absorption coefficients for the diketo and keto-enol forms in the isochoric acetonitrile/water solvent were approximately 13.8 × 10^7^ and 3.6 × 10^7^ L/mol, respectively. The obtained parameters allowed the absorbance to be converted into AVB molarity.

**Figure 8:**
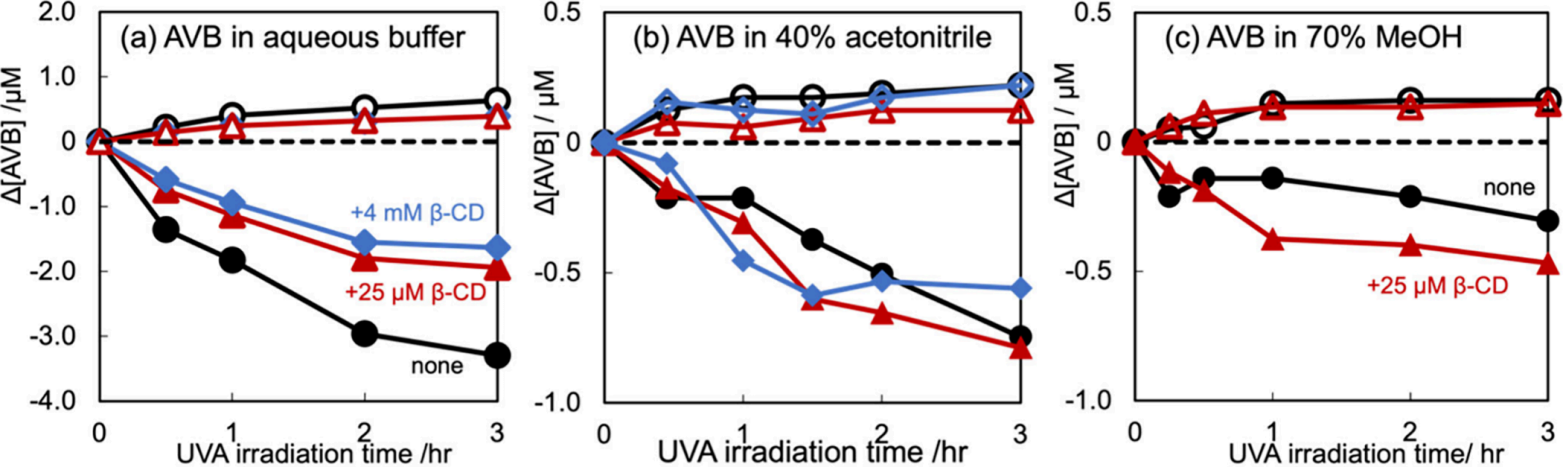
Time evolution of the keto-enol and diketo molarity increase/decrease in the (a) aqueous buffer, (b) 40% acetonitrile, and (c) 70% MeOH upon adding none (circles), equimolar (25 μM, triangles), or excess (4 mM, rhombuses) β-CD. UVA1 (365 nm) irradiation gradually degraded a part of the keto-enol form so that the amount of the keto-enol form (closed signs) descended. Meanwhile, the diketo form (open signs) was elevated. The protecting effect of the equimolar or excess β-CD on the photodegradation seemed equivalent to 40% acetonitrile. The 70% methanol solution experiments were limited, but we obtained similar results within the examinable range.

Figure 8 shows the time course of the keto-enol and diketo molarity increase/decrease in the absence or presence of β-CD. UVA1 (365 nm) irradiation gradually degraded part of the chelated keto-enol form; therefore, this form decreased. In contrast, the diketo form increased. Adding β-CD attenuated the molarity decrease of the chelated keto-enol species of AVB depending upon the amount of β-CD, whereas adding β-CD induced a marginal change in the molarity increase of the diketo species of AVB (Figure 8a). The diketo increase to the keto-enol decrease was 19.3, 20.1, and 23.9% for none, 25 μM β-CD, and 4 mM β-CD, respectively, suggesting that β-CD included the AVB into its internal cavity and converted the chelated keto-enol form to the diketo form. In this study, because the UVA1 lamp induced photodegradation, AVB in the diketo form was not photodegraded.

The protecting effect of equimolar or excess β-CD on the AVB photodegradation appeared equivalent to that of 40% acetonitrile, and less than that of 70% MeOH. The specific permittivities of water, acetonitrile, methanol, and ethanol are 80.0, 37.5, 33.3, and 25.0, respectively.^94^ Predictions based on the mixed solvents owing to a simple linear combination afford specific permittivity values for 40% acetonitrile, 70% methanol, and 30% ethanol of 62.8, 47.3, and 63.5, respectively. The apparent permittivity corresponding to the internal cavity of β-CD would be comparable to these values. Figure S11 suggests that the molar ratio of keto-enol and the diketo species in the presence of β-CD corresponds to that between those in 40 and 50% acetonitrile and is less than that in 70% methanol.

According to Mturi and Martincigh^87^ and Henson et al.,^33^ UV irradiation does not affect AVB in dry methanol, which is a protic solvent. This suggests that excess methanol inhibited AVB photodegradation. The solvent permittivity did not precisely evaluate the hydrophobicity of the internal cavity of β-CD. Topological arguments for the percolation theory or kissing numbers suggest that intermolecular hydrogen bonding between AVB and solvents regulates the conformational or tautomeric rearrangements and the related photoreactivity of the keto-enol moiety. The photostability study conducted by Kumari et al.^93^ would be apt, as their use of 30% protic ethanol and the use of 40% aprotic acetonitrile in this study stabilized AVB to the same level as the internal cavity of the inclusion host.

Conclusively, the hydrophobicity in the internal cavity of β-CD is comparable to that in the 40–50% acetonitrile aqueous solvent.

### 3.8 Additional Discussion

The photostability of AVB under solar light irradiation showed no significant difference compared to UVA1 irradiation, whereas OXB and CUR became destabilized. Because most UVB in solar light is absorbed by the ozone layer, only a small amount reaches the Earth’s surface. OXB is sensitive to UVB, and this small fraction of UVB may cause its degradation. According to the data in this study, the degradation of CUR under UVA1 irradiation was milder than that observed under solar light irradiation. Yan et al.^95^ reported that CUR undergoes degradation 1.7 times faster under 425 nm light irradiation compared to 620 nm light irradiation. On the basis of these findings, it is possible that the solar light accelerated the degradation of CUR.

The solubilization of the CD-inclusion complexes involves equilibrated solubility and dissolution kinetics.^47−49,60^ In this study, we obtained irregular phase-solubility diagrams, contrary to the Higuchi–Connors catalog^45,46^ (see Figure 2). Previously, we discussed the relationship between the solid state of the ligand and the CD inclusion complex as observed by X-ray powder diffraction patterns, time courses of dissolution in aqueous solution, and phase-solubility diagrams.^49^ The programmed mixing ratio can accomplish the spring (i.e., fast dissolution) and parachute (i.e., stabilization of the supersaturated drug solution) effects.^21,50,60^

Although the inclusion complex of AVB and β-CD in the solid state was considered to form the guest and host ratio of 1:2,^69,70^ its phase-solubility diagram was similar to that of the B_S_-type, indicating a molar ratio of 1:1 in solution at less than 5 mM β-CD. In this case, the proportionally increasing phase in a low concentration of β-CD provided the equimolar complex; the plateau phase in ≥5 mM of β-CD induced the production of the 1:2 complex. Discrepancies may exist between the dissolved and solid states (aggregation). The solubility of the equimolar complex is higher than that of the 1:2 complex. This could be the reason the highest concentration in the proportionally increasing phase was higher than the plateau level of the apparent solubility of AVB.

Although the OXB/β-CD complex with a ratio of 1:2 has not been reported in the literature, to the best of our knowledge, the phase-solubility diagram of OXB to β-CD resulted in a similar curve. Plausibly, the plateau level would correspond to the formation of the 2:2 or more equimolar complex, of which the solubility could be lower than that of the equimolar OXB/β-CD complex generated in the proportionally increasing phase. Kumari et al.^96^ claimed a tandem supramolecular structure with equimolar complexes of guest OXB and the cyclic host molecule *C*-methylresorcin[4]arene. The researchers observed an unceasing drift in the NMR spectral chemical shift when the proportion of this host was 0–8 times that of the guest. The equimolar complex was not at the saturation point. Loftsson et al.^97^ stated that aqueous α-CD, β-CD, and γ-CD that contain poorly soluble drugs form microparticles with diameters ranging between 1 and 50 μm at relatively high CD concentrations. Aqueous parenteral solutions of HP-β-CD at a relatively high concentration sometimes aggregate as transient clusters (or transient particulate matter) with a diameter greater than 1000 μm.^96^

Adding β-CD beyond the proportional increasing phase for AVB and β-CD enables the AVB/β-CD complex to form microparticle aggregations. If the β-CD concentration is more than 40–100 times that of the AVB concentration, the greater the increase in the 267 and 360 nm peaks, as shown in Figure 4a. Excess β-CD induces the corresponding diketo and chelated keto-enol species^98^ included in β-CD lamination. The chelated keto-enol species is the most stable isomer, which absorbs the UVA1 band owing to π–π* transitions.^87^ In contrast, the diketo species is a metastable isomer in which intramolecular hydrogen bonding is cleaved and the conjugation of the keto-enol bridge is lost to eliminate the protective effect of UVA1 irradiation.^99,100^ Instead, this species absorbs the UVC band due to the *n*−π* transitions to generate the triplet state, which induces an electronic transition to a singlet state; the cleavage of the α-carbon produces two radical species owing to a Norrish type I reaction.^101^ Therefore, a valid way to improve the photostability and efficacy of AVB would be to transfer the excitation energy of the diketo species to any other quencher.^102^ OCX and OCR, which absorb UVB and are poorly biodegradable, have achieved excellent results as candidate quenchers.^102^

In this study, the hyperchromism of the 267 nm peak was more dominant than that of the 360 nm peak. As the diketone species is relatively hydrophilic, the environment in the internal cavity of β-CD favors including the diketo species instead of the chelated keto-enol species with hydrophobicity. To identify the solvent with hydrophobicity, polarity, or permittivity equivalent to those of the internal cavity of β-CD, 70% methanol (protic) and various proportions of acetonitrile (aprotic) aqueous solutions were used. Because the UV–vis spectral pattern, peak wavelengths, and molar extinction coefficient of AVB vary depending upon the solvent components,^33,87^ the molar populations of the chelated keto-enol and diketo species were evaluated using the signal integral intensity in the NMR spectra.

In dry acetonitrile, the molar proportion of the relatively hydrophilic diketo species was approximately 1:50, and that of the hydrophobic chelated keto-enol species was the most significant. Increasing the water content led to an increase in the proportion of the diketo species, and the obvious UV–vis spectral pattern in the 40% acetonitrile aqueous solution became consistent with that of the AVB/β-CD complex in an aqueous solution. In the 40% acetonitrile aqueous solution, the effect of UVA1 irradiation on AVB was suppressed in the absence and presence of β-CD, as shown in Figure 8b. The 70% methanol solution experiments were limited; however, similar results were obtained within the examined range. Kumari et al.^93^ reported a fundamental investigation using a similar approach, which resulted in a 30–40% ethanol solution providing an equivalent atmosphere to that of the internal cavity of β-CD.

The internal cavity of β-CD retains the favorable hydrophobicity to induce hypochromicity for AVB, but excess β-CD induces hyperchromicity because of aggregation to form microparticles. When β-CD was used to improve the solubility of AVB, β-CD with an appropriate ratio of 40–100 times to AVB favors inducing photostability in AVB. An improvement in AVB solubility enables a decrease in the transdermal transfer of this hydrophobic ingredient to the blood. A local anesthetic, dibucaine, protects the analgesic ketoprofen from photodegradation,^26^ but β-CD accelerates its photodegradation.^27^ Although CD inclusion complexes generally improve aqueous solubility, chemical and metabolic stabilization, unpleasant smell, and taste, cases in which CDs enhance decomposition, defeasance, and disablement do occur. From β-CD, AVB suppresses UVA1 absorption and transforms into a diketo species that is not degradable by UVA1 irradiation. In this study, we did not verify the general disadvantage of the low photostability of AVB;^33^ therefore, these observations cannot contribute to commercially available sunscreen products. This study has illustrated the storage of diketo species in the internal cavity of β-CD.

## 4 Conclusion

According to Avdeef’s diagram, AVB, OXB, and CUR have log *P* values of 4.27, 2.99, and 2.65, respectively, and p*K*_a_ values of 8.59, 7.97, and 8.81, respectively. The phase-solubility diagrams for AVB/β-CD and OXB/β-CD showed a proportional increase at less than 5–6 mM β-CD and plateaued at higher concentrations. Adding β-CD resulted in a hypochromic effect at less than 2 mM β-CD and a hyperchromic effect at ≥2 mM β-CD in the UV–vis spectra of AVB. However, this did not affect the OXB spectrum. At low β-CD concentrations, the hydrophobic internal cavity of β-CD reduced AVB absorbance. At ≥2 mM β-CD concentration, the AVB/β-CD complex aggregated as soluble particles, and the spectral pattern was magnified. The hypothermic effect at the 388 nm shoulder to 360 nm peak bordered on 2 mM β-CD to 50 μM AVB. This reflects the formation of intramolecular hydrogen bonds in the keto-enol moiety of the AVB tautomer in the hydrophobic cavity. These experimental observations suggest that the hydrophobic cavity of β-CD protects against AVB photodegradation by balancing keto-enol and diketo tautomerism. We artificially prepared an appropriate hydrophobic atmosphere for AVB and observed AVB photostabilization in a 40–50% acetonitrile aqueous solution. The molar ratio 1:50–100 of AVB and β-CD is optimum for photostabilization. The proposed condition to apply β-CD to the sunscreen AVB was expected to be efficient in solubilizing and avoiding harmful transdermal permeation into blood and organs.

## Supporting Information Available

SVD analysis for the UV–vis spectra of AVB under UVA1 irradiation in the absence and presence of β-CD (Introduction), RP-HPLC chromatograms of AVB under UVA1 irradiation in the absence and presence of β-CD (Figure S1), RP-HPLC chromatogram of neat CUR (Figure S2), UV–vis spectra of drugs irradiated with solar light in the absence and presence β-CD (Figure S3), RP-HPLC chromatograms of CUR under UVA1 irradiation in the absence and presence of β-CD (Figure S4), UV–vis spectra of AVB under UVA1 irradiation in the absence or presence of β-CD (Figure S5), UV–vis spectra of OXB under UVA1 irradiation in the absence or presence of β-CD (Figure S6), UV–vis spectra of CUR under UVA1 irradiation in the absence or presence of β-CD (Figure S7), ^1^H NMR spectrum of AVB in the methanol-*d*_4_/D_2_O = 7:3 solvent (Figure S8), ^1^H NMR spectrum of AVB in the acetonitrile-*d*_3_/D_2_O = 1:1 solvent (Figure S9), ^1^H NMR spectrum of AVB/β-CD equimolar mixture in the acetonitrile-*d*_3_/D_2_O = 1:1 solvent (Figure S10), NMR titration for keto-enol and diketo molar ratio (Figure S11), results of UV–vis measurements of AVB in methanol/H_2_O = 7:3 solvent (Figure S12), UV–vis spectra of AVB in acetonitrile/H_2_O = 1:1 solvent (Figure S13), and three-dimensional plot of the trajectory analysis results for UV–vis spectra of AVB under UVA1 irradiation in the absence and presence of β-CD (Appendix) (PDF)
Three-dimensional plot of the trajectory analysis results for UV–vis spectra of AVB under UVA1 irradiation in the absence and presence of β-CD (Video) (MP4)

## Abbreviations Used

AVB: avobenzone
BMDM: *p*-*tert*-butyl-*p*-methoxydibenzoylmethane
BP: benzophenone
CD: cyclodextrin
CUR: curcumin
DMSO: dimethyl sulfoxide
U.S. FDA: United States Food and Drug Administration
GRASE: generally recognized as safe and effective
HMBP: 2-hydroxy-4-methoxyphenylbenzophenone
HP-β-CD: hydroxypropyl-β-CD
JP: Japanese Pharmacopoeia
OCR: octocrylene
OCX: octinoxate
OTC: over the counter
OXB: oxybenzone
PABA: *para*-aminobenzoic acid
SBE-β-CD: sulfobutyl ether-β-CD
UV: ultraviolet
