# Supplementary material for: Stability and Properties of Ultraviolet Filter Avobenzone under Its Diketo/Enol Tautomerization Induced by Molecular Encapsulation with β-Cyclodextrin
Source: Langmuir. 2025 Jan 7;41(2):1429–45. doi: 10.1021/acs.langmuir.4c04108 (PMC11755785; doi:10.1021/acs.langmuir.4c04108)
Supplement: Supplementary file 1 — la4c04108_si_001.pdf [file la4c04108_si_001.pdf]

Supporting Information

Stability and properties of ultraviolet filter avobenzone under its diketo/enol tautomerization induced by molecular encapsulation with *β*-cyclodextrin

Chihiro Kuroda[1¶]; Tomohiro Tsuchida[1¶]; Chihiro Tsunoda[1]; Megumi Minamide[1]; Ryosuke Hiroshige[1]; and Satoru Goto[1*]

[1]Faculty of Pharmaceutical Sciences, Tokyo University of Science,

2641 Yamazaki, Noda, Chiba 278-8510, Japan

[¶]These authors contributed equally to this work as the co-first author.

*Correspondence to: Satoru Goto. E-mail: s.510@rs.tus.ac.jp

The provided supporting information includes:

INTRODUCTION: SVD analysis for the UV-Vis spectra of AVB under UVA1 irradiation in the absence and presence of β-CD.

Figure S1: RP-HPLC chromatograms of AVB under UVA1 irradiation in the absence and presence of β-CD.

Figure S2: RP-HPLC chromatogram of neat CUR.

Figure S3. UV-Vis spectra of drugs irradiated with solar light in the absence and presence β-CD.

Figure S4: RP-HPLC chromatograms of CUR under UVA1 irradiation in the absence and presence of β-CD.

Figure S5: UV-Vis spectra of AVB under UVA1 irradiation in the absence or presence of β-CD.

Figure S6: UV-Vis spectra of OXB under UVA1 irradiation in the absence or presence of β-CD.

Figure S7: UV-Vis spectra of CUR under UVA1 irradiation in the absence or presence of β-CD.

Figure S8: H^1-NMR spectrum of AVB in the methanol-d_4:D_2O=7:3 solvent.

Figure S9: ^1H-NMR spectrum of AVB in the acetonitrile-d_3:D_2O=1:1 solvent.

Figure S10: ^1H-NMR spectrum of AVB/β-CD equimolar mixture in the acetonitrile-d_3:D_2O=1:1 solvent.

Figure S11: NMR titration for keto-enol and diketo molar ratio.

Figure S12: The results of UV-Vis measurements of AVB in methanol:H_2O=7:3 solvent.

Figure S13: UV-Vis spectra of AVB in acetonitrile:H_2O=1:1 solvent.

APPENDIX: Three-dimensional plot of the trajectory analysis results for UV-Vis spectra of AVB under UVA1 irradiation in the absence and presence of β-CD.

INTRODUCTION

The observed spectrum for a single sample consists of the column vector, which was scanned for 230-700 nm at an interval of 1 nm (*m*=471). The matrix M for the obtained spectral data is a horizontally sequential row (*n*=64) of the column vectors for all samples illustrated in Figures 3a, 4b, 4c, 3d, and 4a.

The singular value decomposition (SVD) computation decomposes the M to derive the product of three matrices: the rectangular matrix Ψ containing the basis vectors (basis functions) ψ_i, the diagonal matrix Σ consisting of the singular values σ_i in descending order, and the transposed rectangular matrix Λ^t for the singular vectors λ_j. The first basis function reveals the averaged spectrum for all samples, and the continuous basis functions represent the differential spectra depending on the experimental conditions for measurements. The observed spectra are reproduced/approximated by the linear combinations of the basis functions multiplied by the corresponding singular values and singular vectors.

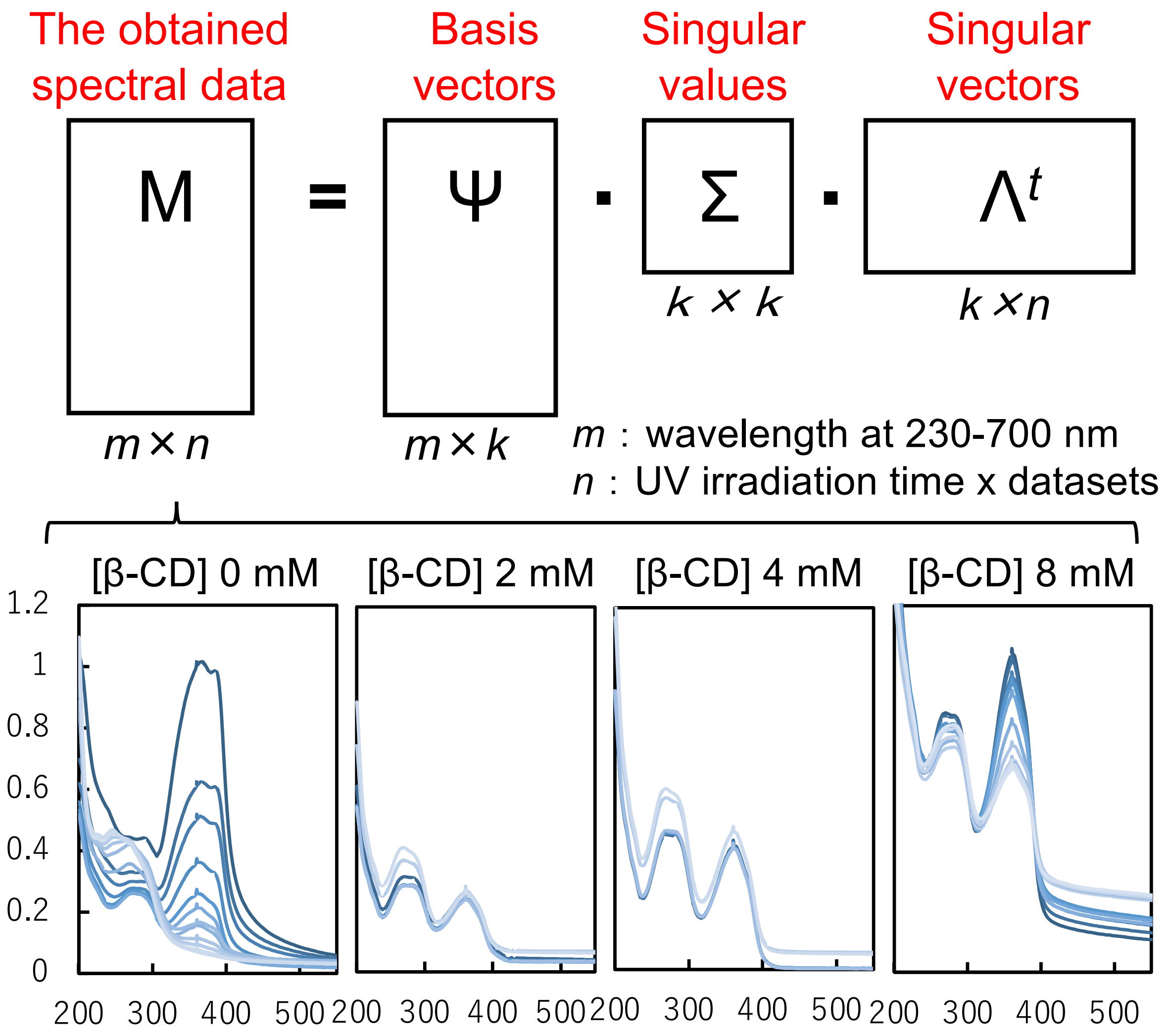

The SVD computation provided the basis functions ψ_i, singular values σ_i, and singular vectors λ_i. Rank is a label of the observed sample, being less or equal to *n*=64. Singular values σ_i are in descending order, and the largest value corresponds to the average spectrum. Statistically, the cumulative amount of singular values σ_i represents the variance in reproduction for the approximated spectrum. the relative cumulative value for the third singular value σ_3 was 89.9%, and that for the fourth singular value σ_4 was 94.4%, leading to the rank of 3 or 4 sufficient to reproduction. The spectra extracted as the basis functions were demonstrated in right panel. The first basis function (blue) is flip-flapped, representing the average spectrum containing 360 and 270 nm peaks. The second basis function (amber) has a positive peak at 270 nm and a negative peak at 393 nm, which seems to reflect morphing during the AVB photodegradation without β-CD, as shown in Figure 3a. The third basis function (gray) has negative peaks at 270 and 366 nm, corresponding to a proportion of the 270 and 360 nm peak heights. The fourth basis function (yellow) seems strange to connect to the actual spectral drifts in the observed data. As it would not accord to the continuous change during the UV irradiation, we considered that indicating the difference among the experimental spectral series.

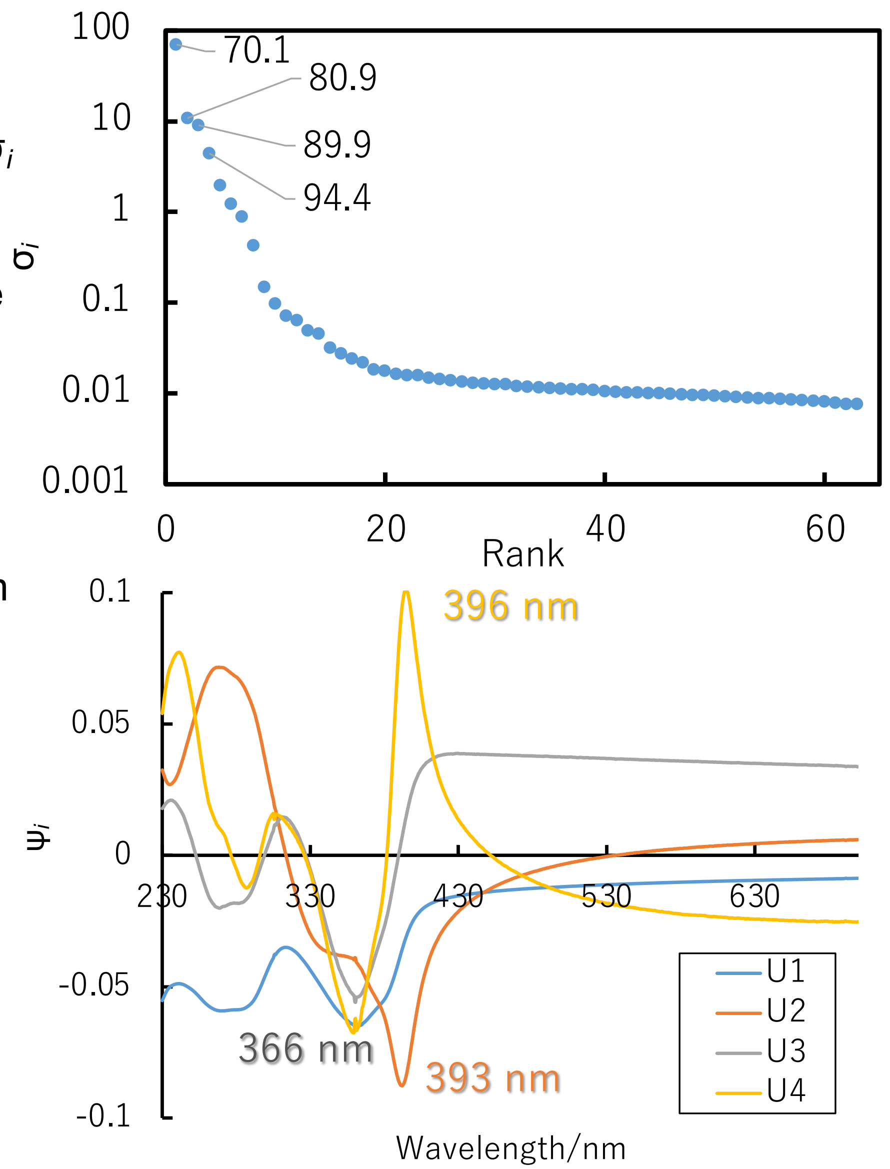

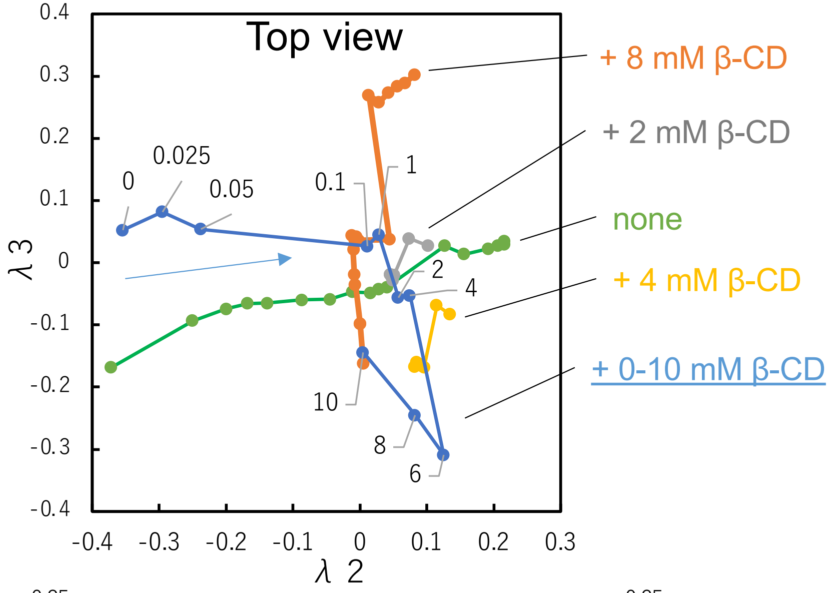

The 3D manifold projected onto the three-sided 2D diagrams represents trajectory analysis of the singular vectors for the composition at ranks 2, 3, and 4. The λ_2-axis represents the spectral change depending on UVA1 irradiation time (green) shown in Figure 3a. Simultaneously, the λ_4-axis indicates the peak height, following the spectral changes after a day. The λ_3-axis reflected the spectral change of the AVB inclusion complex with β-CD (amber) due to UV irradiation, as shown in Figure 3d. Depending on the AVB concentration, the 360 nm peak immediately decreased, and the 267 nm peak was relatively dominant. The amber trajectory for AVB spectra with an excess 8 mM β-CD was along the λ_3-axis to a negative direction, corresponding to the descending 360 nm peak.

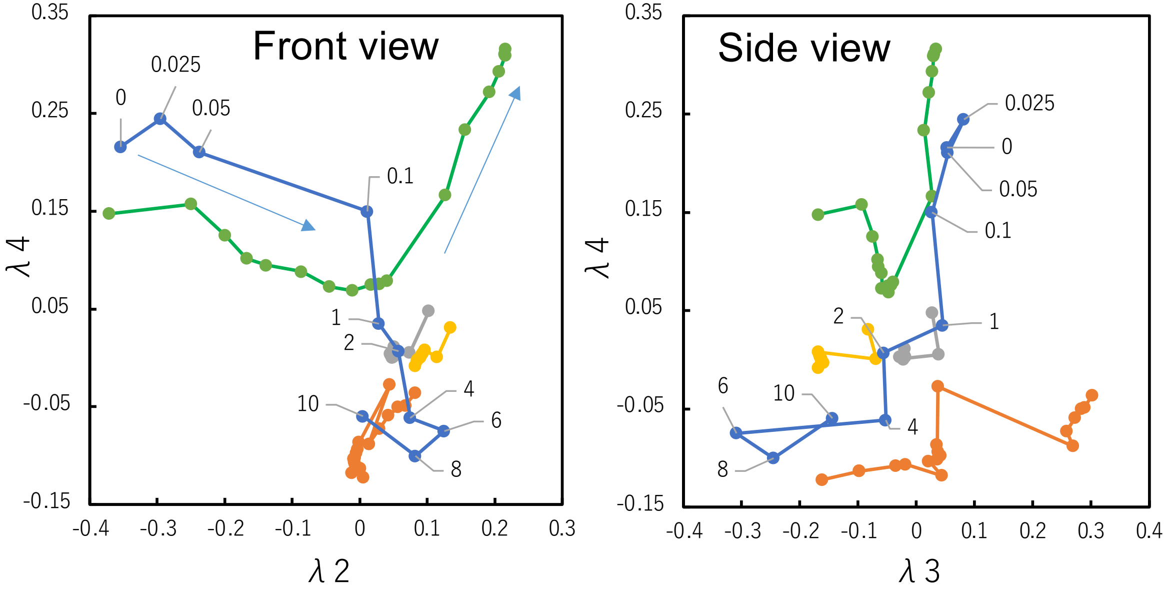

The spectra of the β-CD concentration (blue) had a direction resembling the green trajectory, and the lower β-CD concentration followed the green one. At 0.1-1 mM β-CD, this positive trajectory along the λ_2-axis turns to the negative direction along the λ_3- and λ_4-axes to a negative direction continued in higher concentrations. The fluctuation in the trajectories of photodegradation in the presence of 2 mM or 4 mM β-CD was insignificant. So, we concluded that the 2-4 mM β-CD provides protection against any spectral changes. (The 3D projection movie is available.)

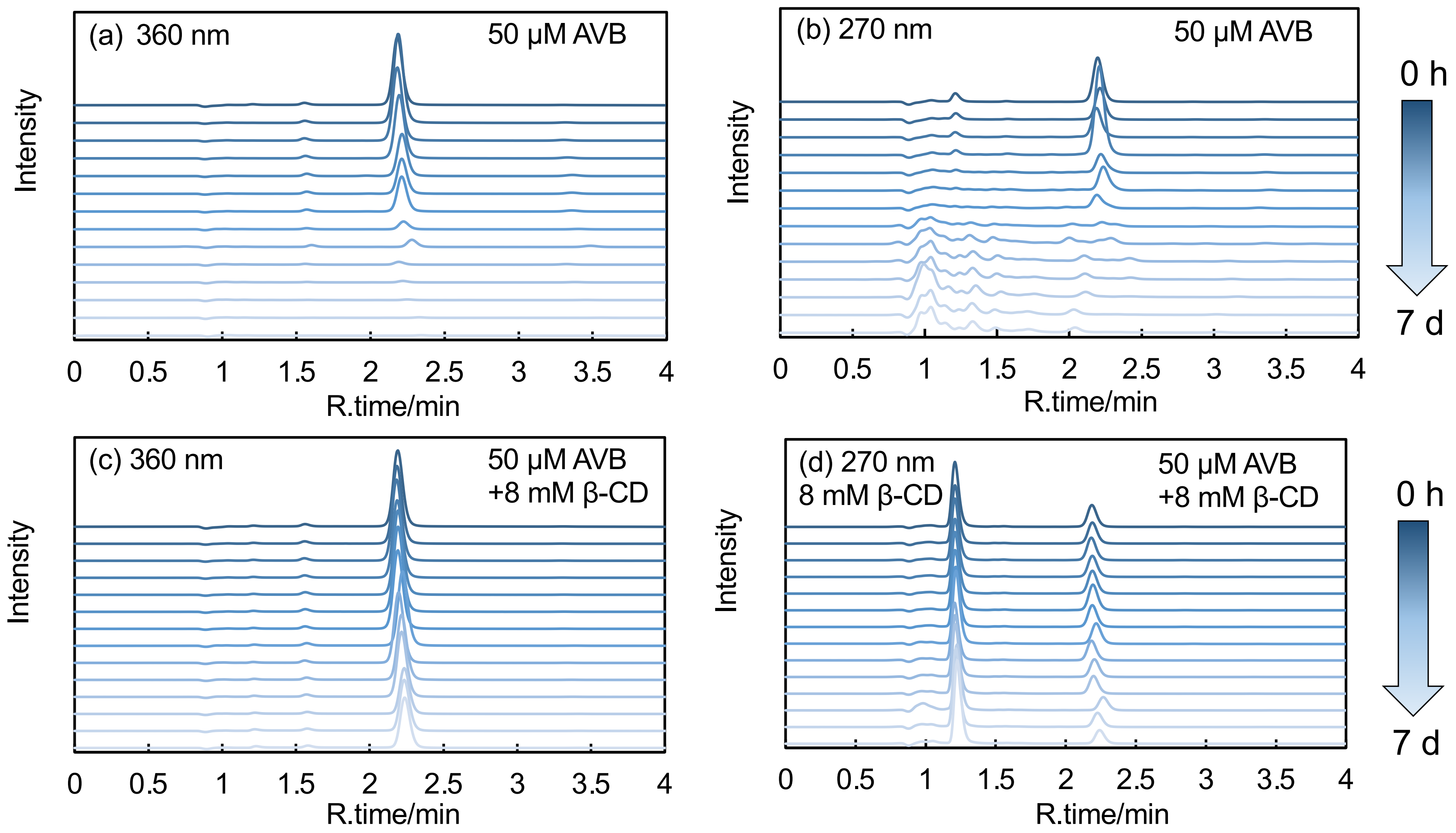

Figure S1. RP-HPLC chromatograms of 50 µM AVB under UVA1 (365 nm) irradiation in the absence (a: at 360 nm and b: at 270 nm) and presence (c: at 360 nm and d: at 270 nm) of 8 mM β-CD in 25 mM phosphate/NaOH buffer (pH 6.8). The chart traces were obtained at 0, 1/5, 1/2, 1, 2, 3, 4, 24, 48 (2 d), 72 (3 d), 96 (4 d), 120 (5 d), 144 (6 d), and 168 (7 d) hours.

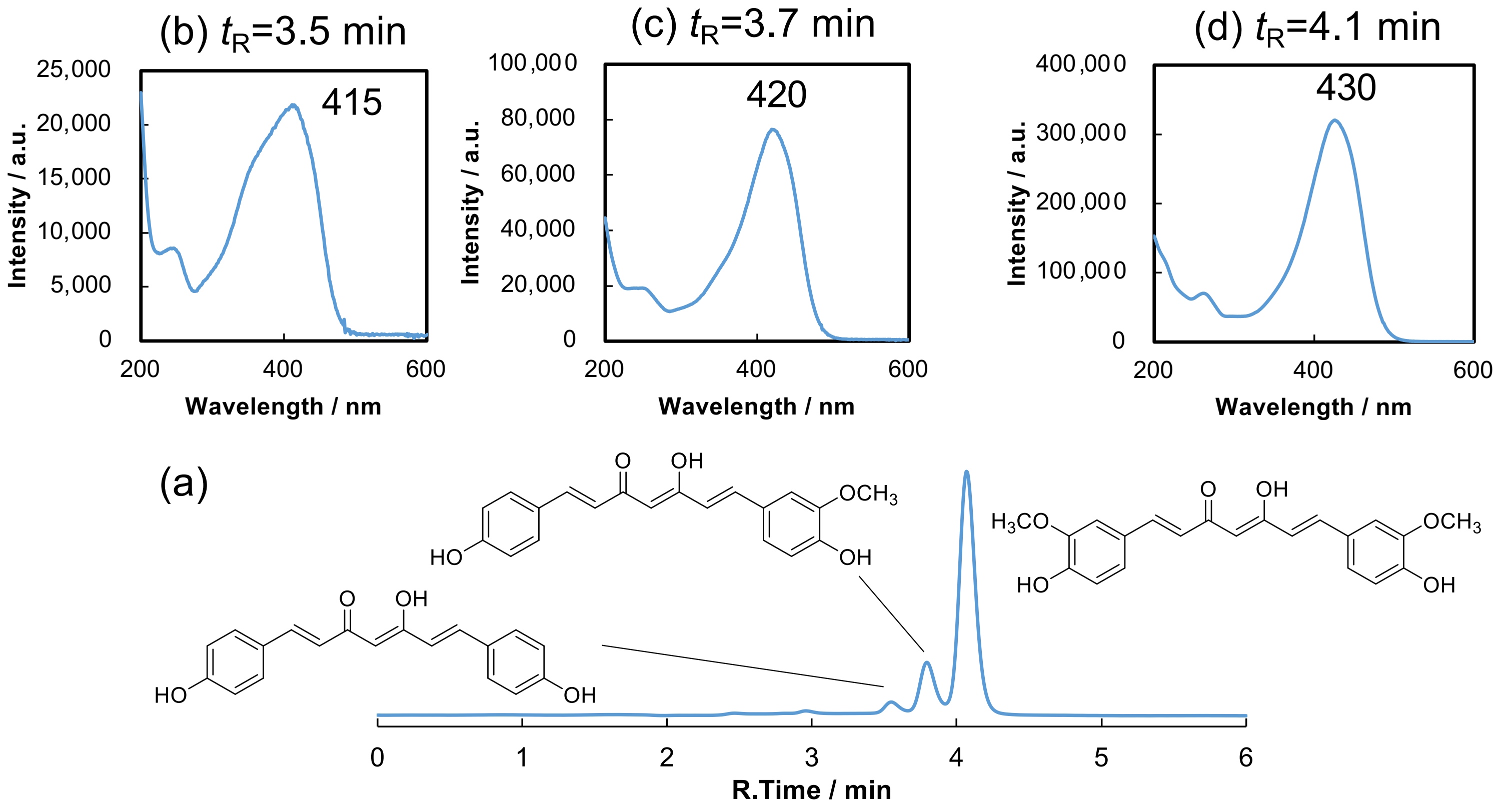

Figure S2. (a) Reversed-phase HPLC chromatogram of neat CUR. Stationary and mobile phases were the ODS column (150 by ϕ4.6 mm) and 25 mM phosphate/NaOH buffer(pH 2.5) in 40% acetonitrile, respectively. Signals were observed with the photodiode array (PDA) at 200-600 nm wavelength. (b), (c), and (d) represented the cross-sections of spectra at the retention times t_R of 3.5, 3.7 and 4.1 minutes, respectively. Didemethoxyl curcumin, demethoxyl curcumin, and curcumin were with the absorption peaks at 415, 420, and 430 nm, respectively. In the measurements for CUR, the tautomerized species were not detected at different retention times in RP-HPLC analyses.

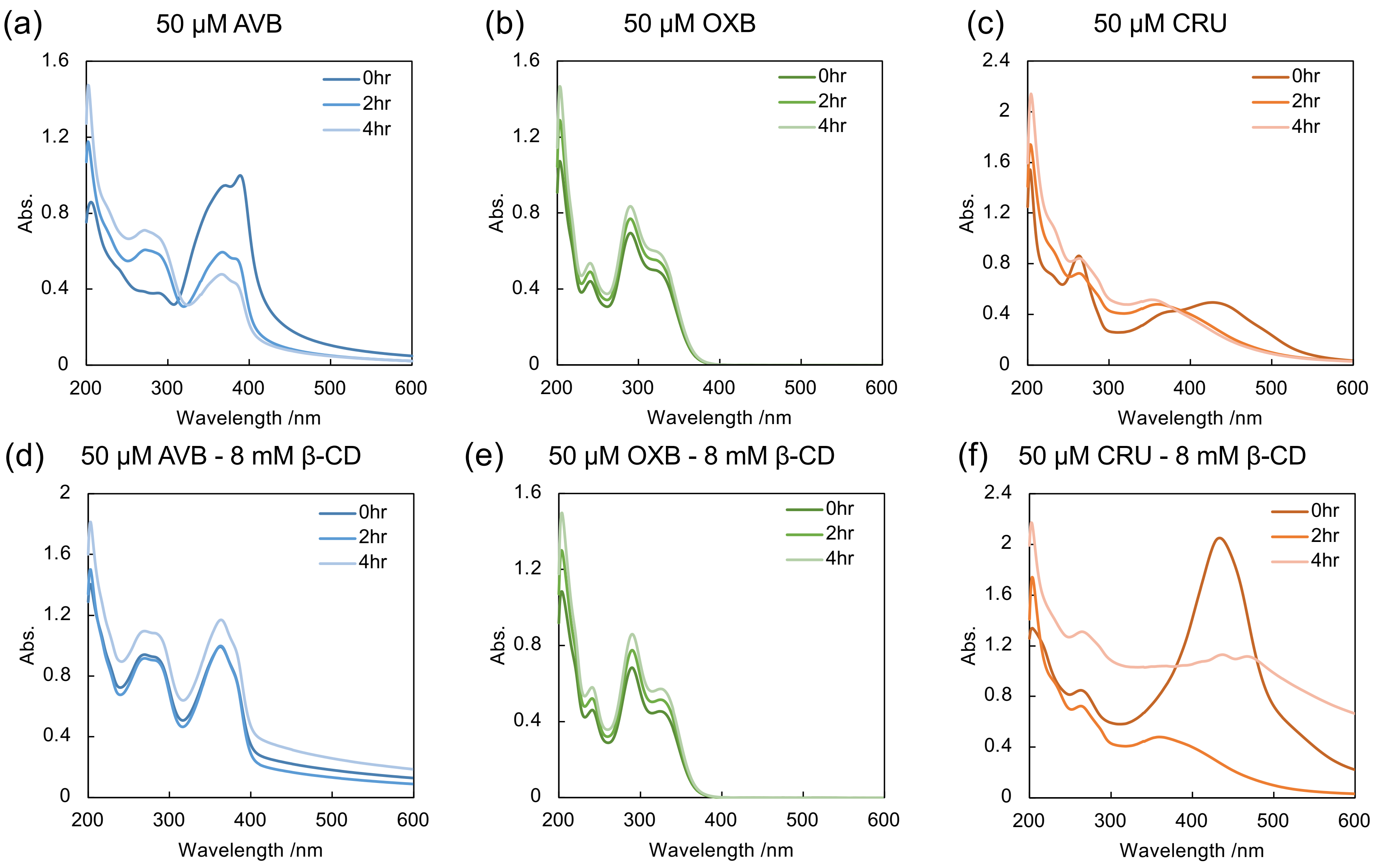

Figure S3. UV-Vis spectra of (a) 50 μM AVB, (b) OXB, and (c) CRU irradiated with solar light in 25 mM Tris-HCl (pH 7.4). The spectra (d)-(f) correspond to these drugs irradiated with the solar light in 8 mM β-CD.

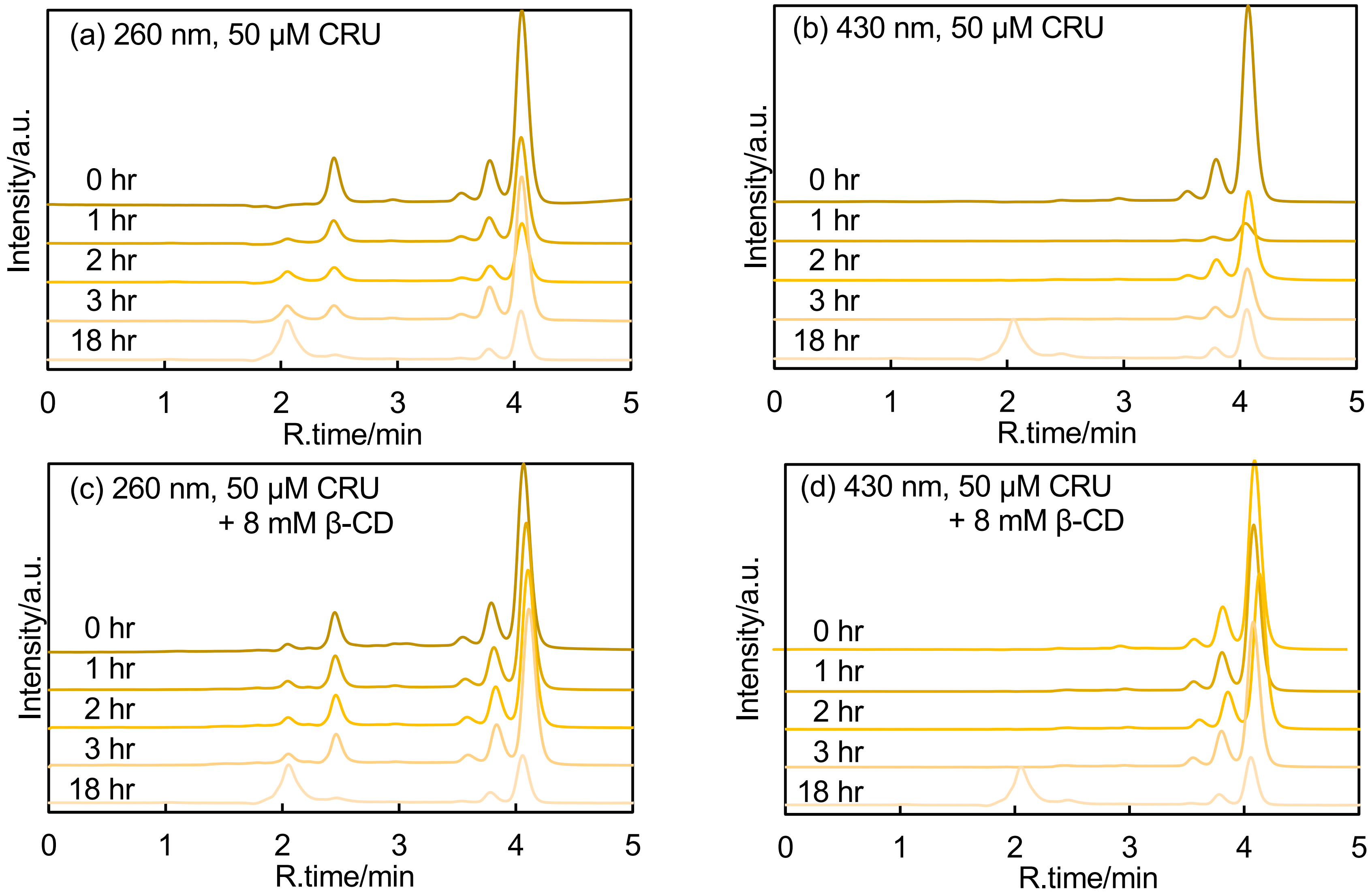

Figure S4. RP-HPLC chromatograms of 50 μM CUR under UVA1 (365 nm) irradiation in the absence (a: at 260 nm and b: at 430 nm) and presence (c: at 260 nm and d: at 432 nm) of 8 mM β-CD in 25 mM phosphate/NaOH buffer (pH 6.8).

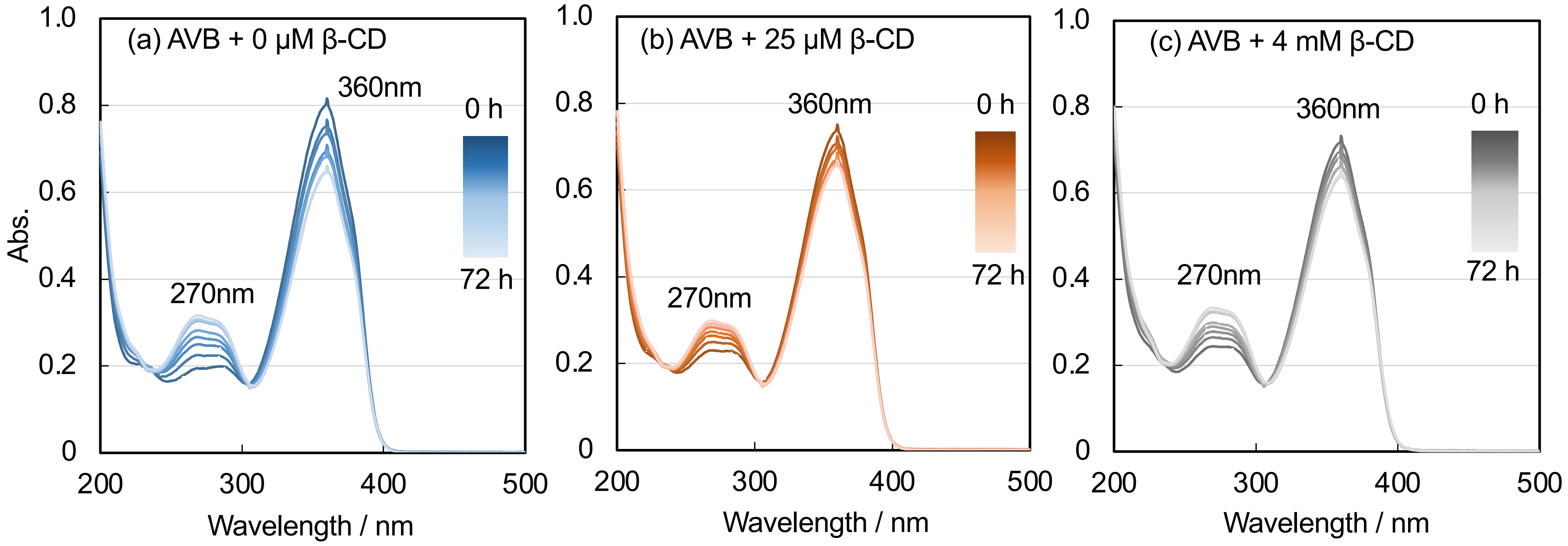

Figure S5. UV-Vis spectra of 25 μM AVB under UVA1 (365 nm) irradiation for 0-72 hours in the absence or presence of β-CD in H_2O:acetonitrile=1:1. (a) AVB alone, (b) in presence of 25 μM β-CD, (c) in presence of 4 mM β-CD.

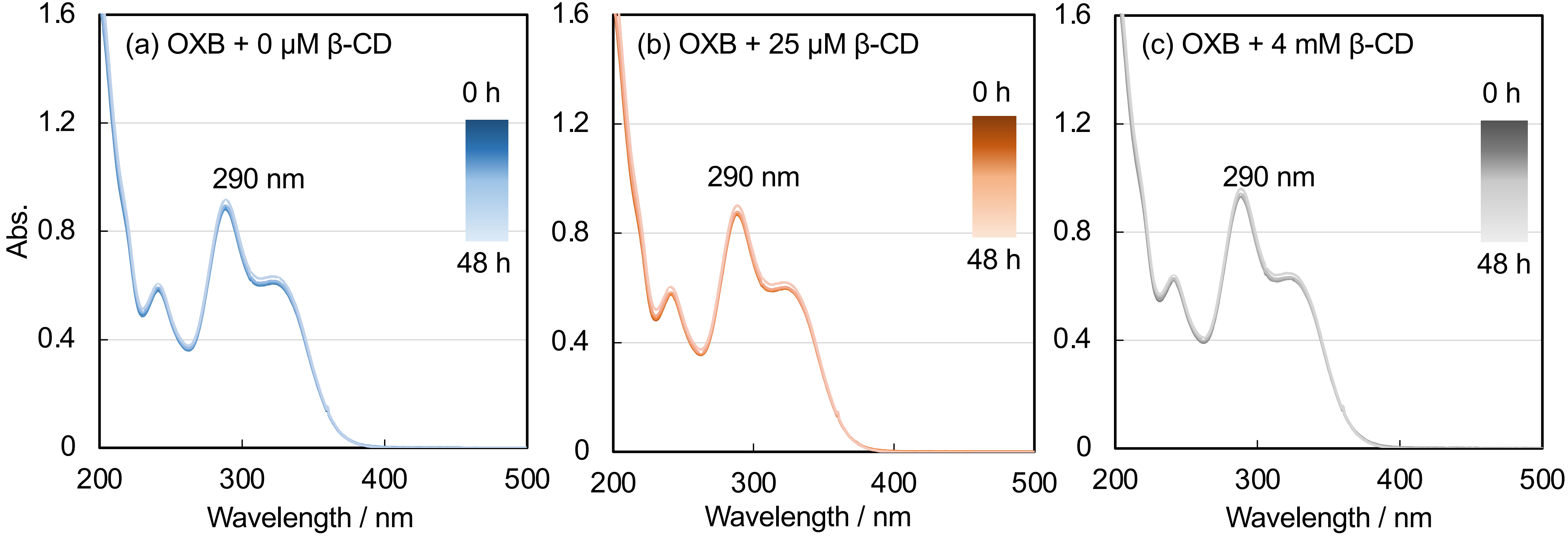

Figure S6. UV-Vis spectra of 25 μM OXB under UVA1 (365 nm) irradiation for 0-72 hours in the absence or presence of β-CD in H_2O:acetonitrile=1:1. (a) OXB alone, (b) in presence of 25 μM β-CD, (c) in presence of 4 mM β-CD.

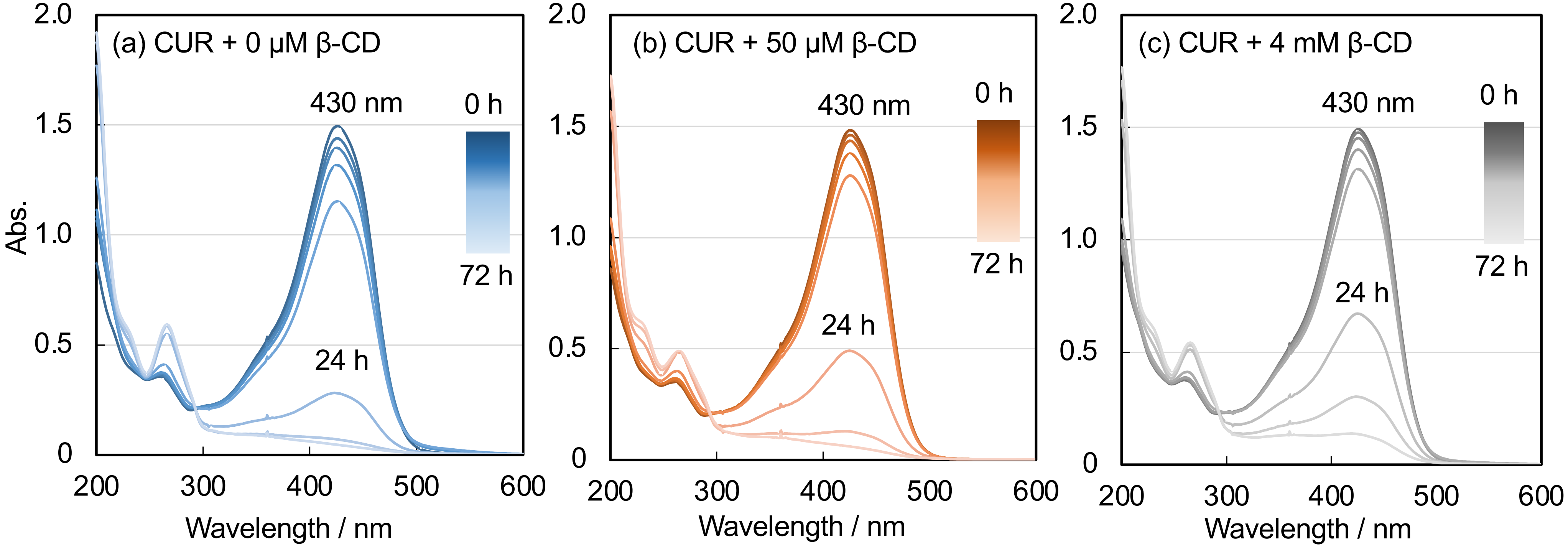

Figure S7. UV-Vis spectra of 25 μM CUR under UVA1 (365 nm) irradiation for 0-72 hours in the absence or presence of β-CD in H_2O:acetonitrile=1:1. (a) CUR alone, (b) in presence of 25 μM β-CD, (c) in presence of 4 mM β-CD.

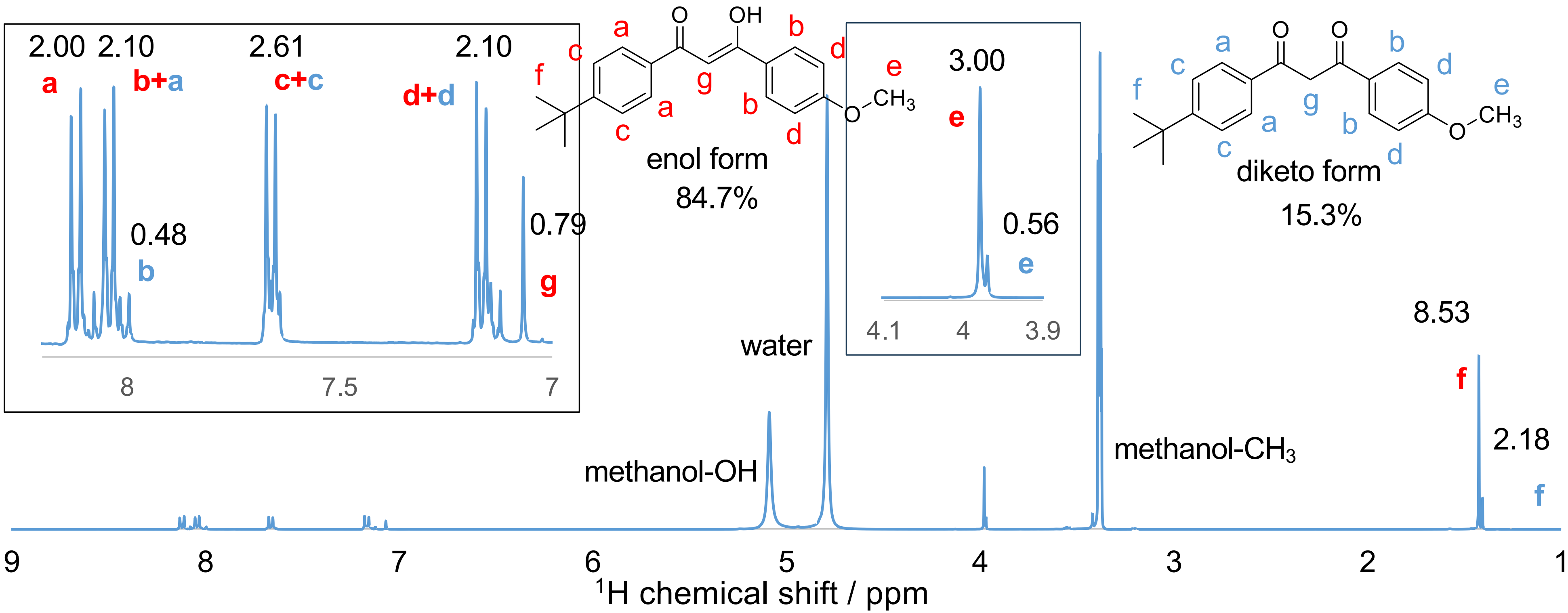

Figure S8. The 400 MHz ^{1}H-NMR spectrum of AVB in the methanol-d_4:D_2O=7:3 solvent. Chemical shift was calibrated at 0 ppm with the tetramethylsilane and 4.790 ppm with the water signal as internal standard. The integrations were calibrated as two for *ortho*-protons of the *p*-*tert*-butylbenzoyl moiety in the enol form. The insets were enlarged views around the a-d and e signals. Hydroxyl proton was reported to be assigned at 17.05 ppm in chloroform-d_1 but not observed. The signals of enol aliphatic and hydroxyl protons of enol form was not detected. The enol and diketo forms ratio was calculated as averages of the integration values as displayed.

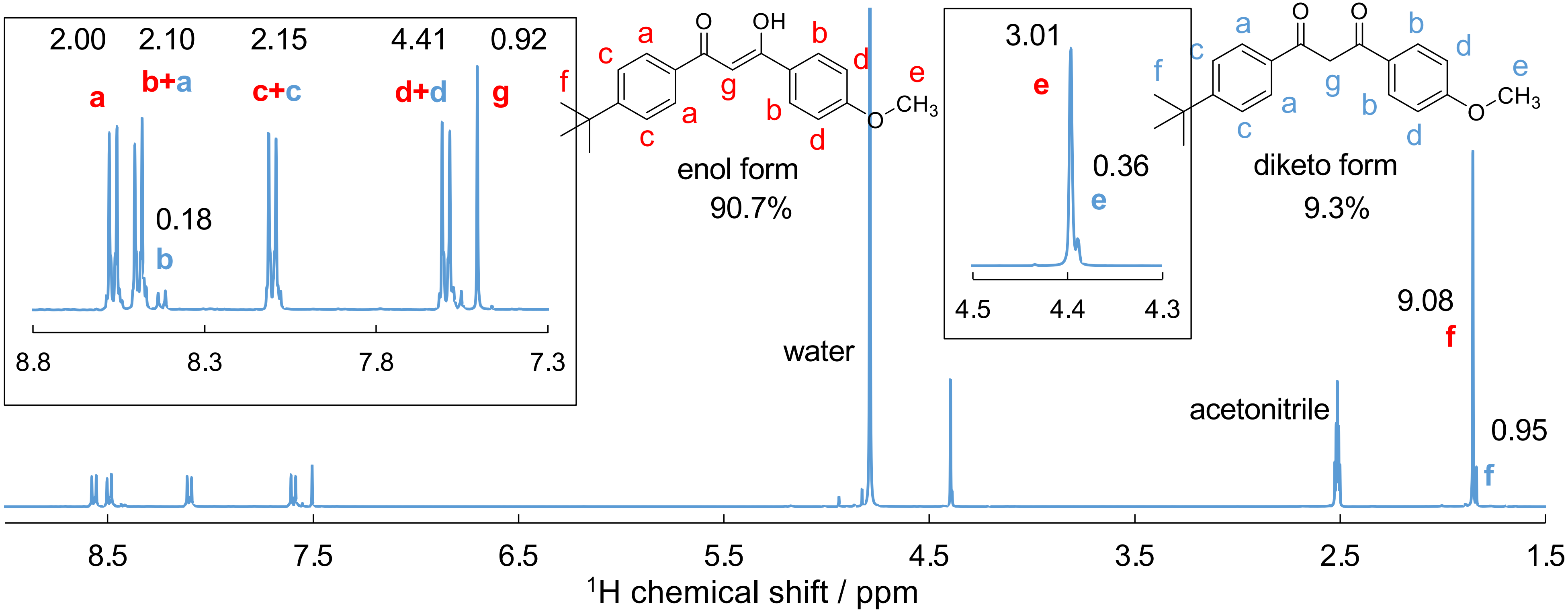

Figure S9. The 400 MHz ^{1}H-NMR spectrum of AVB in the acetonitrile-d_3:D_2O=1:1 solvent. The chemical shift was calibrated at 0 ppm with the tetramethyl silane and 4.790 ppm with the water signal as an internal standard. The integrations were calibrated as two for *ortho*-protons of the *p*-*tert*-butylbenzoyl moiety in the enol form. The insets were enlarged views around the a-d and e signals. Hydroxyl proton was reported to be assigned at 17.05 ppm in chloroform-d_1 but not observed. The signals of enol aliphatic and hydroxyl protons of keto-enol form were not detected. The keto-enol and diketo forms ratio was calculated as averages of the integration values as displayed.

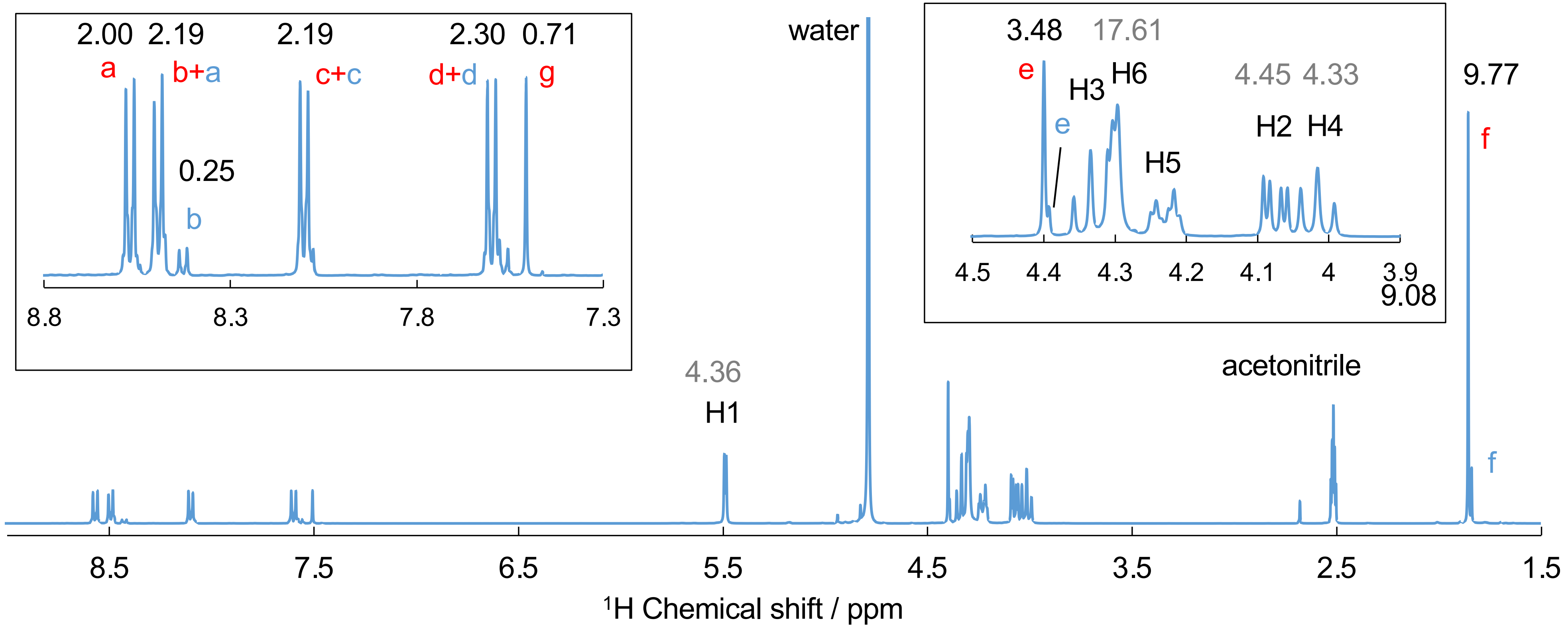

Figure S10. The 400 MHz ^{1}H-NMR spectrum of AVB/β-CD equimolar mixture in the acetonitrile-d_3:D_2O=1:1 solvent. Chemical shift was calibrated at 0 ppm with the tetramethylsilane and 4.790 ppm with the water signal as internal standard. The integrations were calibrated as two for *ortho*-protons of the *p*-*tert*-butylbenzoyl moiety in the enol form. The signals of β-CD were assigned as the singlet at 5.486 ppm for anomeric H1, the triplet at 4.333 ppm for H3, the doublet at 4.298 ppm for H6, the multiplet at 4.230 ppm for H5, the double doublet at 4.070 ppm for H2, and the triplet at 4.015 ppm for H4.

		β-CD	Acetonitrile v/v%				Methanol v/v%
			40	50	75	100	70
Population (%)	Enol	–	79.9	90.7	96.5	98.0	84.7
	Keto		20.1	9.3	3.5	2.0	15.3
	Enol	+	-	87.4	-	-	-
	Keto		-	12.6	-	-	-

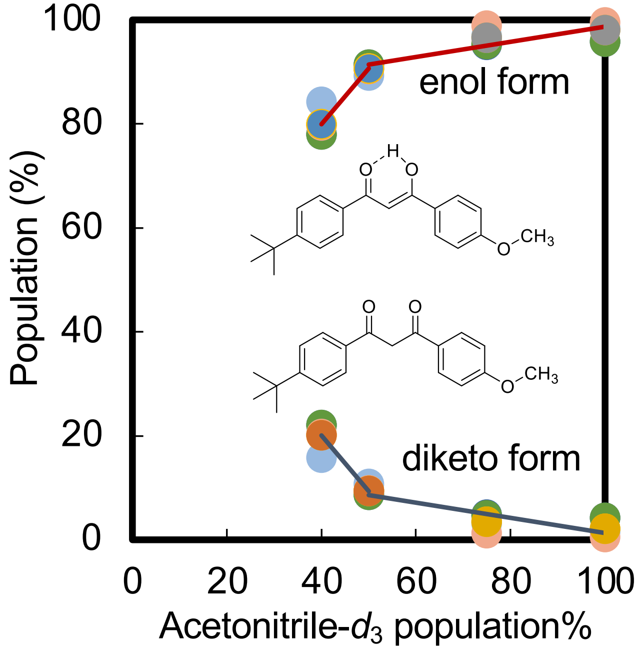

Figure S11. NMR titration for keto-enol and diketo molar ratio in the acetonitrile-d_3/D_2O and methanol-d_4/D_2O solvents. Details for the NMR measurements and analyses were described in the legends of Figures S7, S8, and S9. The populations of the keto-enol and diketo forms were calculated from the isolated signals for the protons a, b, e, and f. The diketo form population in 40% acetonitrile-d_3 became 1/5, while that in 100% acetonitrile-d_3 was 1/50. The keto-enol form population decreased, the diketo form population increased, and their absolute gradients became gentle, depending on the population of acetonitrile in the solvent mixture.

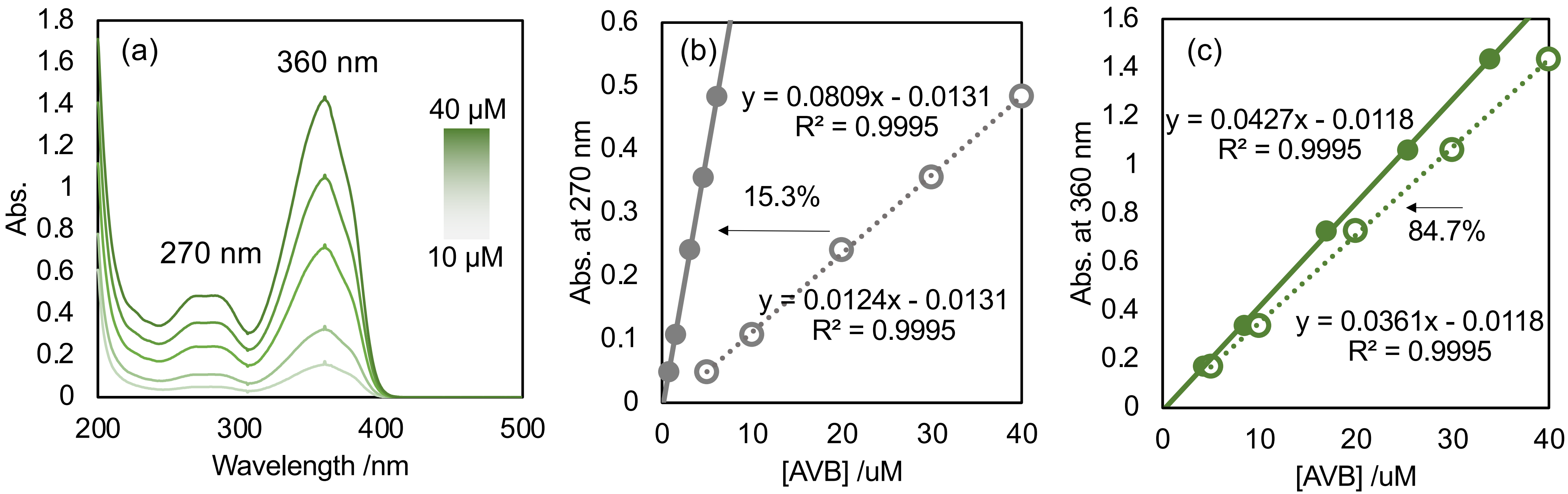

Figure S12. UV-Vis spectra of 10-40 μM AVB in methanol:H_2O=7:3 solvent (a), the regression lines (calibration lines) of absorbance at 270 nm (b), and 360 nm (c). According to the NMR titration, the diketo and enol forms were expected to be consisted at the proportions of 15.3% and 84.7%, respectively. Therefore, the abscissae of the regression lines (open signs) were condensed to the species contents (closed signs), indicating that the molar absorption coefficients of the diketo and enol forms were about 8.1×10^7 and 4.3×10^7 L/mol.

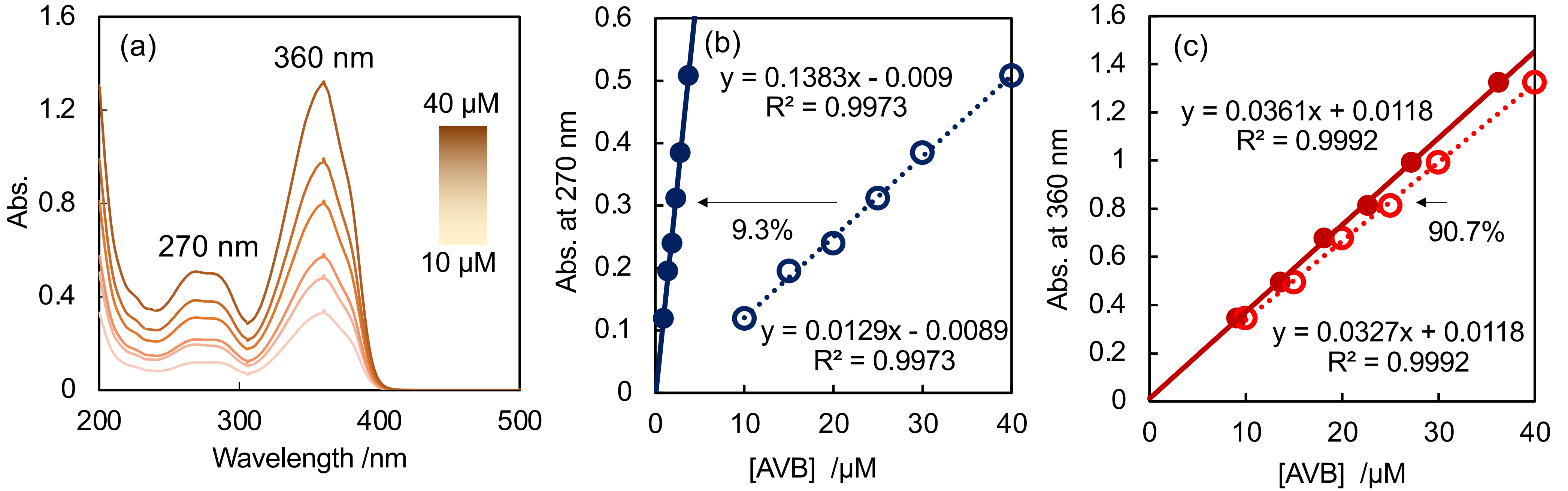

Figure S13. UV-Vis spectra of 10-40 μM AVB in acetonitrile:H_2O=1:1 solvent (a), the regression lines (calibration lines) of absorbance at 270 nm (b), and 360 nm (c). According to the NMR titration, the diketo and enol forms were expected to be consisted at the proportions of 9.3% and 90.7%, respectively. Therefore, the abscissae of the regression lines (open signs) were condensed to the species contents (closed signs), indicating that the molar absorption coefficients of the diketo and enol forms were about 13.8×10^7 and 3.6×10^7 L/mol.

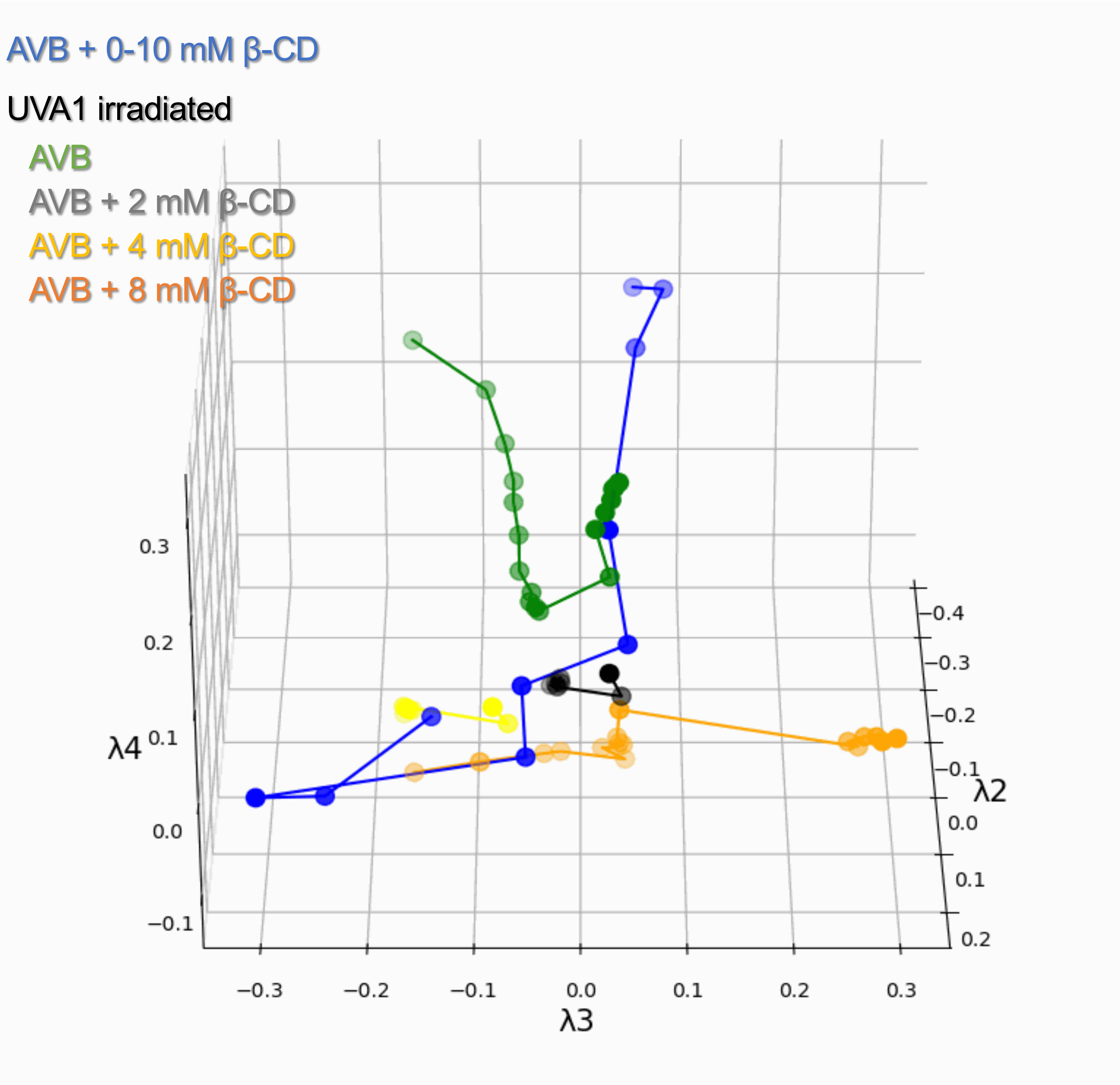

APPENDIX

(1) Upon adding 0-10 mM β-CD, the blue trajectory initially followed the escalating direction along the λ2-axis and bent to the descending direction along the λ4-axis at around λ2=0. Further, it changed to the descending direction along the λ3-axis step by step and looped at the highest concentration. Compared to Figure 4a, the increment along the λ2-axis corresponded to the descending 388 nm peak, and the decrement along the λ3-axis represented the enhancement of 267 and 360 nm peaks. The last turning indicated the balance between the heights of 267 and 360 nm peaks.

(2) The green trajectory for UVA1-irradiated neat AVB followed almost parallel to the blue one. At the λ2=0, it kept increasing along the λ2-axis and simultaneously elevated along the λ4-axis. According to Figure 3a, the λ4-axis corresponded to the increment of 267 nm peak.

(3) The amber trajectory expressed UVA1-irradiated AVB with excess β-CD, moving along the λ3-axis. Figure 3d shows the decrement of the 267 and 360 nm peaks and the switch of the dominance of the former to the latter, supporting that the λ3-axis represented the balance of the heights of 267 and 360 nm peaks.

(4) Adding 2 or 4 mM β-CD shrank the spectral changes of AVB during UVA1 irradiation.
